# Long-Gap Sciatic Nerve Regeneration Using 3D-Printed Nerve Conduits with Controlled FGF‑2 Release

**DOI:** 10.1021/acsami.5c08237

**Published:** 2025-07-07

**Authors:** Diego N. Rodriguez-Sanchez, Leticia A. M. de Carvalho, Ingri Mancilla-Corzo, Luciana P. Cartarozzi, Saeed Safari, Menekse Ermis, Marcos A. d’Ávila, Alexandre L. R. Oliveira

**Affiliations:** † Laboratory of Nerve Regeneration, Department of Structural and Functional Biology, Institute of Biology, University of Campinas (UNICAMP), Campinas, Sao Paulo 13083-970, Brazil; ‡ 678581 Terasaki Institute for Biomedical Innovation (TIBI), Woodland Hills, California 91367, United States; § Department of Manufacturing and Materials Engineering, School of Mechanical Engineering, 28132 University of Campinas (UNICAMP), Campinas, Sao Paulo 13083-860, Brazil

**Keywords:** nerve guidance conduits, peripheral nerve regeneration, biomaterials, 3D printing, FGF-2, gelatin methacryloyl, polycaprolactone

## Abstract

Peripheral nerve injuries (PNIs) by transection require reconstructive surgery, often with highly variable results and persistent sensory and motor deficits. Three-dimensional (3D) printing enables the biofabrication of nerve guidance conduits (NGCs) with the ability to release neurotrophic factors, showing therapeutic potential. We developed a 3D printing process of NGCs using polycaprolactone (PCL) and gelatin methacryloyl (GelMA) integrated with a thermostable fibroblast growth factor 2 (FGF-2). The synthesized GelMA at 10% (w/v) concentration showed superior rheological, mechanical, and ultrastructural characteristics, ensuring 3D printing fidelity. Incorporating FGF-2 into GelMA resulted in a controlled release pattern over 30 days along with biocompatibility and an increase of metabolism in rat S16 Schwann cells and human mesenchymal stem cells (MSCs). MSCs exhibited gene regulation linked to vascularization after FGF-2 stimulation. The PCL polymer facilitated the buildability of a spiral-patterned tubular structure, which was functionalized with a combination of GelMA and UV photocrosslinked. At 12 weeks, following a long-gap nerve injury in rats, NGC implantation enhanced sensory and motor recovery, improved electrophysiological function, and promoted morphological and ultrastructural nerve reorganization and regeneration. At 4 weeks, significant Schwann cell proliferation (S100), expression of the pan-neurotrophin receptor (P75NTR), myelination of newly grown axons, and organization of neurofilaments were observed. The bioactive NGCs represent a promising alternative to nerve autografts for the repair of long-gap injuries.

## Graphical abstract

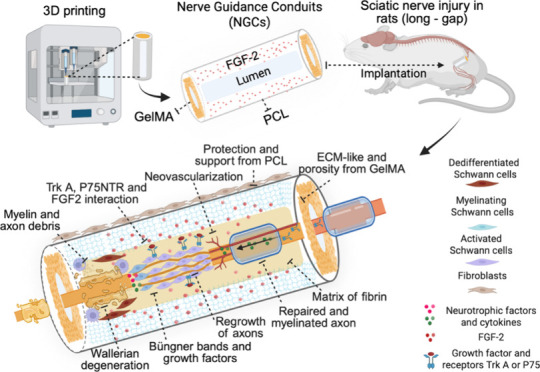

## Introduction

Peripheral nervous system injuries (PNIs) affect 22 million people in developed countries with an incidence of 4.38 per 100,000 inhabitants per year and an estimated annual healthcare expense of $150 billion.^1,2^ Compression, traction, and avulsion of the nerves due to open/closed traumas or iatrogenic causes lead to highly debilitating sensory/motor injuries, significantly impacting the quality of life.^1,3^ Studies involving large cohorts showed that active young adult men contributing to the economy are most affected by automobile and machinery accidents, requiring long-lasting rehabilitation.^3^ Two types of injury are observed in these cases: short defects (<8 mm), where nerve transection can be repaired using end-to-end neurorrhaphy,^4,5^ and long defects (>3 cm), where neurorrhaphy cannot be performed without excessive tension, demanding the use of nerve autografts.^5^ These grafts are generally obtained from sensory donor nerves (e.g., from the sural nerve).^6^ However, limitations include incomplete functional recovery, inadequate revascularization, limited donor tissue, prolonged surgical time, and morphological and functional mismatch between nerves.^5,7^ Nerve allograft use may be an alternative but requires immunosuppression.^6^ Despite surgical refinements, a meta-analysis reported that injuries to the ulnar and median nerves showed only 42.6% and 51.6% sensory recovery, respectively.^8^

After nerve transection, a series of regenerative responses are initiated in the proximal and distal nerve segments as well as in neuronal cell bodies.^9,10^ These include the upregulation of regeneration-associated genes (RAGs) and proteins alongside the Wallerian degeneration of distal axons and myelin. This process supports axonal growth across the injury site and aids in the reinnervation of muscles.^9,10^ Transcription factors that regulate proregenerative neurotrophic factors such as nerve growth factor (NGF), brain-derived neurotrophic factor (BDNF), neurotrophins-3, -4, and -5 (NT-3/4/5), fibroblast growth factor 2 (FGF-2), neuregulins, ciliary neurotrophic factor (CNTF), growth-associated protein 43 (GAP-43), actin, and tubulin are produced in a spatiotemporal way, requiring sustained release especially in severe nerve injuries.^9^ However, chronic and long-segment injuries result in a loss of sustained neurotrophic and structural support, thereby limiting the regenerative capacity of the nerve.^10^ In PNIs with long-gaps, regenerating axons may not receive sufficient neurotrophic support to initiate the sprouting. Additionally, the proliferative capacity and survival of Schwann cells progressively decline, impairing axonal regeneration and remyelination.^11^ Finally, the loss of nerve-muscle connection affects muscle fibers, resulting in severe atrophy.^5^ Although the peak regenerative capacity occurs shortly after injury, the therapeutic window for nerve repair and axonal regeneration is narrow and limited.^9,10^

Fibroblast growth factor (FGF), specifically FGF-2, plays multiple roles and is constitutively expressed *in vivo* in the peripheral nervous system (PNS) and central nervous system (CNS) under physiological conditions.^12^ FGF-2 is critical in promoting functional recovery in the PNS and CNS by supporting neuronal survival, controlling excitotoxicity, promoting axonal regeneration and angiogenesis, reducing apoptosis, and providing neuroprotection.^12,13^ Immobilization of FGF-2 in conventionally fabricated collagen nerve guidance conduits (NGCs) has shown positive results in nerve regeneration following noncritical 5-mm-gap injuries in rodents.^14^ However, neurotrophic factors generally have a short half-life and low stability post-application.^15^ Approaches such as conjugation with heparin, chemical modifications, or genetic engineering for overexpression or microencapsulation have been implemented to improve stability.^15^ Enhancing thermal stability (at 37 °C) using genetically modified FGF-2 mutants and encapsulation in hydrogels that stabilize the protein through ionic interactions are two approaches to enhance the therapeutic effect of trophic factors.^15^

Nerve injury often results in tissue loss and retraction with failure of spontaneous repair. In this context, end-to-end neurorrhaphy without generating tension is not feasible.^5^ Therefore, an alternative approach to autografting is using NGCs to bridge the nerve segments and allow for regeneration.^16–19^ Various synthetic materials [e.g., poly­(glycolic acid), poly­(lactic acid), poly­(ethylene glycol), and polyhydroxybutyrate] or natural materials (e.g., veins, collagen, chitosan, silk, and agarose) have been used to manufacture NGCs using various conventional fabrication techniques.^20–23^ However, these techniques offer simplified architecture and limited choices of materials, dimensions, and porosity.^19,21^ Current NGCs do not promote complete recovery in long defects compared to the autograft technique.^5,23–25^ These limitations have been associated with insufficient Schwann cell migration, low concentration of neurotrophic factors, and inappropriate axonal guidance.^10^ Therefore, modulating nerve regeneration through bioactive, biomimetic biomaterials with controlled release of molecules could be a promising strategy.^19,26–28^

To overcome the above-mentioned limitations, 3D printing technologies involve the organized deposition of biomaterials to create structures based on 3D models with controlled geometry, showing a distinct advantage for nerve construct production.^19,26,29^ Extrusion of hydrogel bioinks is widely used, offering advantages such as the (1) deposition of filaments with multiple diameters, (2) fabrication of highly porous and interconnected structures that facilitate cell growth and nutrient exchange, (3) bioprinting of formulations containing cells, molecules, and biomaterials, and (4) excellent cost-effectiveness with repeatability and scalability.^19,29,30^ Previous studies manufactured 3D-printed hollow NGCs using synthetic materials.^22,23,29,31^ However, combining natural platforms with synthetic biomaterials to tune the release of bioactive molecules or cells could optimize nerve regeneration.^27,28,30^ Functionalization can be achieved using cellular systems (e.g., stem cells and Schwann cells), bioactive molecules, nanozymes, electrically conductive compounds, and drugs.^16,17,23,27,28^

The efficacy of 3D bioprinting depends on adequate mechanical, biochemical, rheological, and biocompatible properties of the biomaterials.^19,30^ Based on the mechanical properties of synthetic polymers (e.g., polycaprolactone, PCL) and the excellent biological properties of natural biomaterials (e.g., gelatin), functional scaffolds can be fabricated for tissue engineering.^31–33^ Gelatin is suitable for cell adhesion, with adaptable physical properties that can be achieved by adding methacrylate groups (gelatin methacryloyl, GelMA).^33–35^ Highly porous and biocompatible constructs based on GelMA fabricated through 3D bioprinting allow for the incorporation of cellular systems, trophic factors, or drugs.^36,37^ PCL polymer is a thermosensitive synthetic polyester with multiple applications in tissue engineering, including 3D printing of NGCs.^16,18,19,31^ GelMA exhibits low mechanical strength and rapid degradation, requiring chemical cross-linking, photocrosslinking, or blending with synthetic polymers such as PCL to overcome these limitations.

In the present work, we developed a 3D printing process for the fabrication of bilayer NGCs using the hybrid approach consisting of an outer wall of PCL and an inner wall of cross-linked GelMA with thermostable FGF-2. We hypothesized that the integration of biomaterials could generate a hybrid system with controlled release of FGF-2 to improve functional and motor recovery in rats subjected to sciatic nerve tubulization with a long-gap between stumps.

## Materials and Methods

### Materials

The reagents used included porcine skin type A gelatin (300 bloom from porcine skin, Sigma-Aldrich), methacrylic anhydride, 1-[4-(2-hydroxyethoxy)­phenyl]-2-hydroxy-2-methyl-1-propan-1-one (Irgacure 2959, Sigma-Aldrich), Corning mini bioreactor (Sigma-Aldrich), and ninhydrin (all from Sigma-Aldrich, Sao Paulo, Brazil), PCL medium-molecular weight (*M*_n_ = 50000; Cellink-BICO, Gothenburg, Sweden), and cellulose dialysis membrane 12–14 kDa (Spectrum Laboratories, Rancho Dominguez, CA). Cell culture and biological characterization: S16 cells were derived from rat sciatic nerve (ATCC CRL-2941, Manassas, VA), poly­(l-lysine) (Sigma-Aldrich, St. Louis, MO), Dulbecco’s modified eagle medium (DMEM) modified to contain 4 mM l-glutamine and 4500 mg/dL glucose (ATCC 30-2202, Manassas, VA), supplemented with 10% fetal bovine serum (FBS; Gibco) and 1% penicillin/streptomycin (Gibco). Cells were treated with Trypsin-EDTA in Hank’s balanced salt solution (HBSS; ATCC 30-2101, Manassas, VA).

### Gelatin Methacryloyl (GelMA) Synthesis

The synthesis of the GelMA precursor solution was performed by diluting 10% (w/v) gelatin in Dulbecco’s phosphate-buffered saline (DPBS). Subsequently, 0.6 g of methacrylic anhydride per 1 g of dissolved gelatin (0.6:1 wt/wt ratio) was added to react for 60 min at 50 °C, as previously described,^35^ with modifications. The solution was diluted with two volumes of ultrapure deionized water (40 °C) and dialyzed against distilled water using dialysis tubes with a cutoff of 12–14 kDa during 1 week at 40 °C. After the purification step, the pH was adjusted to 7.4, and the solution was filtered, frozen in liquid nitrogen, and lyophilized (Lio101, Liobras, Sao Carlos, Brazil) for a week. Finally, the obtained GelMA foam was stored at −80 °C, protected from light and moisture.

### Rheological Analysis

Rheological measurements were performed using a rotational modular rheometer (Anton Paar MCR-102, Austria) with a 50-mm-diameter cone–plate geometry, a cone angle of 0.9815°, and a truncation of 0.97 m. All samples of GelMA hydrogel at 2.5%, 5%, and 10% were deposited on the plate to completely fill the gap (1 mm size) between the plate and cone. Steady-state shear viscosity and shear stress measurements were conducted by varying the shear rate from 1 to 100 s^–1^ using a rotational test at 22 °C. The viscosity was measured over a temperature ramp from 10 to 35 °C at a rate of 1 °C min^–1^, with a shear rate of 1 s^–1^. The storage modulus (*G*′) and loss modulus (*G*″) were measured as a function of temperature at a constant frequency of 1 Hz and a constant strain of 0.1%, which is within the linear viscoelastic regime. The hydrogel samples were equilibrated at 35 °C and then cooled at a rate of 1 °C min^–1^ from 35 to 10 °C (*n* = 2).

To evaluate the viscoelastic behavior of photo-cross-linked GelMA hydrogels at 2.5%, 5%, and 10% (*n* = 2), oscillatory shear stress sweeps in the range of 10^–1^–10^3^ Pa were performed. Also, frequency sweep tests were performed within the linear viscoelastic region (γ_0_ = 1%) in the frequency range of 1–25 s^–1^ at room temperature. Both tests were performed using a rotational modular rheometer (Anton Paar, MCR-102, Austria) using parallel-plate geometry with 25 mm diameter and a 3 mm gap. The hydrogels were cross-linked using round-shaped molds (7 mm diameter and 4 mm height) by exposure to UV light (365 nm, 12 mW cm^–2^, 240 s). Duplicates (*n* = 2) from each concentration were analyzed.

### Scanning Electron Microscopy (SEM)

GelMA hydrogels were freeze-dried for 3 h and mounted onto stubs using double-sided carbon tape. The samples were then coated with gold using a sputter coater (BAL-TEC, SCD-050, Balzers, Liechtenstein) for 200 s. Similarly, PCL scaffolds and 3D-printed NGCs were fixed onto stubs and coated with gold for 200 s. The microstructure of NGCs, GelMA 5%, and 10% hydrogels was examined using a scanning electron microscope (model JSM 5800LV, JEOL, Tokyo, Japan) at 12 kV. Images were captured using *SemAfore 5.21* software (JEOL, Tokyo, Japan). The average pore area (*n* = 4) of the internal wall of 3D-printed 10% GelMA NGCs was analyzed by using the particle analysis tool in *ImageJ*. Additionally, the dimensions of the NGCs, including the wall thickness, internal diameter (ID), and external diameter (ED), were measured. Finally, the printability of the PCL polymer was analyzed using bidimensional 3D-printed scaffolds (*n* = 3), with measurements obtained using *ImageJ* software (NIH, Bethesda, MD).

### Compression Testing

The compressive strength of cross-linked hydrogels was measured at a rate of 2 mm min^–1^ using a mechanical testing machine (Instron 5943, Instron Int. Ltd., Norwood, MA) with *Bluehill*, version 3, software equipped with a 100 N load cell. Triplicates (*n* = 3) from each concentration were analyzed to obtain a compressive stress/extension curve and a compressive modulus.

### Cytocompatibility

The indirect cytotoxicity of GelMA hydrogels (2.5%, 5%, and 10%) was tested in S16 cells derived from a rat sciatic nerve (ATCC CRL-2941, Manassas, VA). *In vitro* metabolism and proliferation were assessed using Presto Blue assay (Thermo Fisher Scientific, A13262) and live/dead assays (Thermo Fisher Scientific, L3224) at 1, 3, and 5 days. Briefly, S16 cells were seeded at a density of 2 × 10^4^ cells cm^–2^ (ATCC CRL-2941, Manassas, VA) in 12-well plates, following the manufacturer’s protocol. The samples were then photo-cross-linked under UV light (18 mW cm^–2^ for 240 s) and immersed in a cell culture medium consisting of DMEM, supplemented with 4 mM l-glutamine, 4500 mg dL^–1^ glucose (ATCC 30-2202, Manassas, VA), 10% FBS (Gibco), and 1% penicillin/streptomycin (Gibco). After incubation for 1, 3, and 5 days, cells were incubated with 1 μg mL^–1^ calcein AM and 2 μg mL^–1^ ethidium homodimer for 30 min. Random images (*n* = 3) were captured using fluorescence microscopy (Echo Revolve, BICO, San Diego, CA). Cells were then treated with 10% Presto Blue for 90 min, and the fluorescence intensity was measured (excitation = 560 nm; emission = 590 nm) using a microplate reader (Varioskan LUX, Thermo Fisher).

### FGF-2 Release Profile

The release of hyperstable FGF-2 from GelMA hydrogels (5% and 10%) was quantified using ELISA. Hydrogels (390 L, 2 μg mL^–1^ FGF-2) were cast in silicone molds (7 × 14 × 4 mm) and UV-cross-linked (18 mW cm^–2^, 240 s). Samples were incubated in 1 mL of release medium (1% BSA in DPBS) at 37 °C while being shaken (60 rpm). Supernatants were collected daily and analyzed per the manufacturer’s protocol (DY233-05, R&D Systems). FGF-2 levels were determined using a standard curve (*n* = 3).

### Gene Expression

Human dental pulp-derived mesenchymal stem cells (MSCs; Poietics, Lonza Walkersville, MD) were plated in triplicate at a density of 2 × 10^5^ cells cm^–2^ in 24-well adherent plates (Costar, 24 wells, Corning, NY) and stimulated with hyperstable FGF-2 at concentrations of 2, 0.2, and 0.02 μg mL^–1^. Cells without stimulation were used as controls. After 72 h in culture, the culture medium was completely aspirated, and the cell monolayer was directly lysed in the plate by adding 350 μL of RLT buffer per well (Qiagen, Sao Paulo, Brazil), followed by vigorous pipetting. The lysate was transferred to a microcentrifuge tube, and any remaining material was collected using a sterile cell scraper. Samples were then stored at −80 °C. RNA was extracted using a silica membrane spin column (Mini RNAeasy kit, Qiagen, Sao Paulo, Brazil) and quantified by spectrophotometry. Complementary DNA (cDNA) synthesis was performed using the High-Capacity cDNA Reverse Transcription Kit (Life Technologies Corp., Carlsbad, CA) following the manufacturer’s instructions. Triplicate reactions for qPCR were conducted using TaqMan assays (Life Technologies Corp., Carlsbad, CA): Hs00900055_m1-VEGF; Hs03805856_g1-BDNF. Samples were tested with the reference gene glyceraldehyde 3-phosphate dehydrogenase (GAPDH). Quantitative qRT-PCR procedures were performed on the MX3005P instrumentation platform (GE Healthcare, Chicago, IL).

### Biofabrication of Functionalized NGCs

To evaluate PCL (*M*_n_ = 50000) printing efficacy and resolution, geometries of 10 mm × 10 mm × 0.5 mm were designed in CAD (computer-aided design) and 3D-printed, and SEM images were obtained. The printability was determined and obtained from PCL scaffolds (*n* = 3), according to ref 38. Additionally, the pore height, pore width, and filament diameter of the PCL constructs (*n* = 3) were evaluated using *ImageJ* software. Subsequently, NGC models, with an internal diameter of 3 mm and a length of 10 mm, were designed using Fusion 360 (Autodesk, San Francisco, CA). The designs were exported in STL format and then sliced by using the DNA Studio bioprinting software (BICO-Cellink, Gothenburg, Sweden). The NGCs were 3D-printed through microextrusion of continuous melted PCL (*M*_n_ = 50000) filaments using a thermoplastic toolhead within a 3D bioprinting setup (Bio X 3 Gen, Cellink-BICO, Gothenburg, Sweden). The printing parameters were as follows: a stainless steel nozzle with a diameter of 300 μm, an extrusion pressure of 180 KPa, a printhead temperature of 180 °C, a printbed temperature of 10 °C, a print velocity of 10 mm s^–1^, a layer height of 0.39 mm (100%), and 0% infill. Postprocessing involved rapid cooling with an external air current, washing three times in DPBS, and sterilization by immersion in 70% ethanol for 30 s, followed by rinsing with DPBS, drying at room temperature, and exposure to UV irradiation (200–280 nm) for 1 h. Then, 50 μl of sterile GelMA 10%/0.5% irgacure 2959 (w/v) hydrogel incorporated with FGF-2 hyperstable (2 μg mL^–1^, Core Biogenesis, Strasbourg, France) was deposited inside the NGCs and subsequently photocrosslinked with UV light (365 nm, 18 mW cm^–2^, 240 s). For this procedure, the NGCs were positioned vertically and secured by using double-sided tape. A guide tube was then inserted into the NGCs, and the GelMA solution was promptly photocross-inked.

### Sciatic Nerve Injury

Female Lewis rats (7 weeks of age) were obtained from the Multidisciplinary Center for Biological Research (CEMIB) at the University of Campinas (UNICAMP). The rats were kept under controlled humidity and temperature conditions with normal light/dark cycles. We adhered to the ethical guidelines established by the National Council for Animal Experimentation Control (CONCEA) and approved by the institutional Ethics Committee on Animal Use (CEUA) (Protocol No. 6180-1/2023). The experimental procedures were performed under anesthesia with isoflurane (Isoforine, Crystalia, Sao Paulo, Brazil) using a microsurgical microscope. Rats underwent critical nerve injury (8 mm of gap) to the sciatic nerve. In the Autograft group, nerves were sectioned proximally and distally, maintaining an 8 mm defect, and subsequently sutured with perineural stitches (10/0 Ethicon, Cincinnati, OH). In the NGCs and NGCs+FGF-2 groups, nerves were injured proximally and distally, creating an 8 mm defect, and then nerve stumps were secured within NGCs using perineural stitches (10/0 Ethicon, Cincinnati, OH). Surgical planes were sutured (5/0 Vycril, Ethicon, Cincinnati, OH), and post-operative tramadol was administered for 5 days. *In vivo* parameters were evaluated in the short (4 weeks) and long (12 weeks) term. Table 1 presents the parameters and groups evaluated.

**Table 1:** Experimental Groups, Number of Animals, and Evaluated Parameters^a^

experimental paradigm	experimental groups	number	time
motor and sensorial analysis	autograft	*n* = 5	long-term (12 weeks)
	NGCs		
	NGCs+FGF2		
electromyography	autograft	*n* = 5	long-term (12 weeks)
	NGCs		
	NGCs+FGF2		
muscle mass	autograft	*n* = 5	long-term (12 weeks)
	NGCs		
	NGCs+FGF2		
immunohistochemistry	autograft	*n* = 3	short-term (4 w)
	NGCs		
	NGCs+FGF2		
morphological analysis	autograft	*n* = 3	long-term (12 weeks)
	NGCs		
	NGCs+FGF2		

a Autograft, autograft group; NGCs, NGCs without FGF-2; NGCs+FGF-2, NGCs with FGF-2, TEM, transmission electron microscopy.

### Motor Functional Evaluation

Motor recovery analysis in the animals was conducted using an automated Catwalk system (Noldus, Wageningen, The Netherlands) with a high-speed video camera (GP-3360, Gevicam, Milpitas, CA) equipped with a wide-angle lens (8.5 mm, Fujicon Corp., Dongguan City, China). Weekly runs were performed for 12 weeks across the autograft, NGCs, and NGCs+FGF2 groups. One baseline motor measurement (prior to injury) was recorded, and four runs per animal were conducted, evaluating the following parameters: peroneal nerve functionality index according to ref 39, as well as the contact area (cm^2^), maximum intensity of contact (average), support area of the thoracic and pelvic limbs (cm^2^), and regularity index (%). The average values were calculated per animal and for each experimental group (*n* = 5).

### Sensory Functional Evaluation

Nociceptive recovery was assessed weekly for 12 weeks in the autograft, NGCs, and NGCs+FGF2 groups. Mechanical sensitivity was evaluated by using the von Frey test. Incremental pressure was applied to the plantar area of the injured limb using a 0.5 mm^2^ polypropylene tip connected to a portable pressure force transducer (Aesthesiometer EFF 301, Ribeirao Preto, Sao Paulo, Brazil) to determine the withdrawal reflex pressure intensity. The hyperalgesia intensity (Δ withdrawal threshold, g) was calculated by subtracting the post-treatment measurements from the first measurement before treatment. The average values were calculated for each experimental group (*n* = 5).

### Electrophysiological Analysis

Electromyography (EMG) measurements were performed at 12 weeks postinjury of the autograft, NGCs, and NGCs+FGF2 groups, including contralateral nerves. Monopolar needle electrodes were placed in the tibial cranial muscle belly to record compound muscle action potentials (CMAPs). Stimulating electrodes were placed directly on sciatic nerve proximal and distal segments of the lesion to generate single supramaximal stimulation. Latency (ms), duration (ms), and amplitude (mV) were displayed using an oscilloscope (TDS3000C, Tektronix, Portland, OR). The motor nerve conduction velocity (NCV) was calculated by dividing the distance between stimulation sites by the average latency evoked from the two sites. The average values were obtained for each experimental group (*n* = 5).

### Muscle Mass

Muscle and body masses were assessed at week 12 in the autograft, NGCs, and NGCs+FGF2 groups. The tibialis cranialis and gastrocnemius muscles were harvested, weighed, and used to determine the total muscle mass (g), which was then compared to the corresponding normal contralateral muscle and between experimental groups. Additionally, the body mass was measured weekly for 12 weeks. The average values were obtained for each experimental group (*n* = 5).

### Immunostaining of the Sciatic Nerve

Immunostaining analysis was conducted 4 weeks after nerve repair in the autograft, NGCs, and NGCs+FGF2 groups. Activation of Schwann cells (S100), cytoskeletal organization (neurofilament), expression of the neurotrophic factor receptor (P75NTR), macrophage activation (IBA-1), and myelination (fluoromyelin) were determined. Following transcardiac perfusion, specimens were collected and divided into three segments: proximal, central, and distal. The central segment (regenerated nerve tissue within NGCs) was fixed in 4% paraformaldehyde in PB (0.1 M, pH 7.4) for 12 h at 4 °C, immersed in sucrose solutions at 10%, 20%, and 30% (0.1 M PB, pH 7.4 for 12 h), and embedded in Tissue-Tek O.C.T (Sakura Finetek, Torrance, CA). Longitudinal cryosections (12 μm) were obtained. During immunostaining, slides were incubated in a blocking solution for 1 h (3% BSA, 0.1 M PB, pH 7.4), followed by primary antibodies for 4 h and secondary antibodies for 45 min. Primary antibodies were S100 (Abcam, AB868, 1:500), Neurofilament H (Millipore, AB1989, 1:200), P75NTR (Santa Cruz, sc-6169, 1:250), IBA-1 (Wako, 019-19741, 1:750), and fluoromyelin stain (Invitrogen, F34651, 1:300). Representative images were captured using a fluorescence microscope (BX51, Olympus Corp., Tokyo, Japan) and quantified using *ImageJ* software (version 1.33u, NIH, Bethesda, MD).

### Morphological Analysis

Morphological analysis by TEM images was performed at week 12 after nerve repair in the autograft, NGCs, and NGCs+FGF2 groups. Perfusion and fixation were performed using Karnovsky’s solution (2% glutaraldehyde and 1% paraformaldehyde in 0.2 M PBS, pH 7.34). The central segment obtained from NGCs was analyzed and divided into two segments: proximal and distal. Subsequently, postfixation was performed with 1% osmium tetroxide diluted in 0.2 M PBS. Fragments were embedded in epoxy resin (Durcupan, Fluka, Sigma-Aldrich) and semithin (450 nm) for morphological analysis, and ultrathin sections (90 nm) were obtained using an ultramicrotome (Ultracut, Leica, Wien, Germany) and collected on copper grids (200 mesh, EMS, Philadelphia, PA). After contrast with uranyl acetate and lead citrate, specimens were observed using the Tecnai G2 Spirit BioTwin transmission electron microscope (FEI, Eindhoven, The Netherlands) operating at 80 kV.

### Statistical Analysis

Quantitative variables were assessed for normality using statistical tests (Shapiro–Wilk or Kolmogorov–Smirnov), descriptive statistics, and graphical analyses (QQ plots). Parametric data underwent analysis of variance (ANOVA, one-way or two-way) followed by Tukey’s posthoc test to compare differences between groups and time points in both *in vitro* and *in vivo* experiments. Nonparametric data were analyzed using the Kruskal–Wallis test. For comparisons between two groups, an unpaired *t* test was used for parametric data, while the Mann–Whitney test was applied for nonparametric data. Statistical significance was defined as *p* < 0.05, with significance levels denoted by asterisks (*p < 0.0332, **p < 0.0021, ***p < 0.0002, and ****p < 0.0001). GraphPad *Prism*, version 9, for Windows was utilized for all statistical analyses.

## Results

### Biomaterial Characterization

Methacryloyl modification of gelatin was analyzed using ninhydrin assay, according to ref 34. The degree of functionalization (DoF) was defined as the difference between this unit value. For example, GelMA samples prepared at 10 mg mL^–1^ reacted with ninhydrin and produced apparent concentrations of 2.59, 2.56, and 2.77 mg mL^–1^ with DoFs of 74.06%, 74.34%, and 72.28, respectively (Figure S1A).

Photocrosslinking assay was performed by exposing 2.5, 5%, and 10% (w/v in PBS) GelMA samples to UV light at 365 nm (18 mW cm^–2^) to define the best parameters (Figure S1B). Samples were prepared with 0.5% irgacure 2959 photoinitiator and cross-linked for 40–300 s. Samples of 2.5% GelMA after UV-light exposure at 40 and 100 s did not showed cross-linking or stability. Therefore, these samples were not included for subsequent time points. Similarly, 5% GelMA exposed for 40 and 100 s exhibited thickening, flowing in the base without complete cross-linking. On the other hand, 10% GelMA showed better structure preservation, vertical stability, and fine edges starting from 100 s on the dorsal view compared to 5% GelMA, which exhibited better cross-linking and resolution above 240 s (Figure S1B). In addition, after an inclination of ∼45°, only 10% GelMA samples were stable (Figure S1C). UV exposure at 18 mW cm^–2^ for 240 s in 10% GelMA was considered to have a superior resolution.

Adjusting the pH to physiological conditions is essential to maintaining cellular homeostasis. After dialysis of GelMA, the pH was adjusted to 7.34, close to physiological levels. After immersion of 2.5%, 5%, and 10% GelMA photocrosslinked gels in a cell culture basal medium, no change in color was noted, showing stable pH. In addition, no significant differences in the pH were observed between the groups at 1, 2, 3, and 5 days post-incubation compared to the control (*p* > 0.05; Figure S1D).

### Rheological Analysis and SEM

The rheological properties of the biomaterial were evaluated in three concentrations of GelMA of 2.5%, 5%, and 10%. Temperature-dependent viscosities are shown in Figure 1A, indicating a reduction of viscosity (pseudoplasticity) during an increase of temperature. In addition, the increase in viscosity was more pronounced at higher GelMA concentrations. A stable thermoresponsive behavior was observed around 20–28 °C across all concentrations (Figure 1A). The pseudoplastic (shear-thinning) behavior was superior in 5% and 10% GelMA after the shear rate was increased compared to the lower concentration of 2.5%. (Figure 1B). In addition, the viscosity was lower at 2.5% and 5% compared to that at the higher 10% at 22 °C (Figure 1B). Figure 1C shows variation of *G*′ and *G*″ of the hydrogels by varying the temperature. The values of the gelation point (*G*′ = *G*″) were ∼24 °C in 2.5% GelMA, ∼27 °C in 5%, and 29 °C in 10%. During cooling, the *G*′ value increased rapidly and crossed over *G*″, indicating the presence of gel-like structures. All concentrations below the gelation point (*G*″ > *G*′) showed a liquid-like behavior. Based on the rheological findings, 5% and 10% GelMA were chosen due to the superior shear-thinning behavior, high storage modulus (*G*′), and gelification above room temperature, allowing subsequent evaluation of the 3D printing capacity.

**Figure 1:**
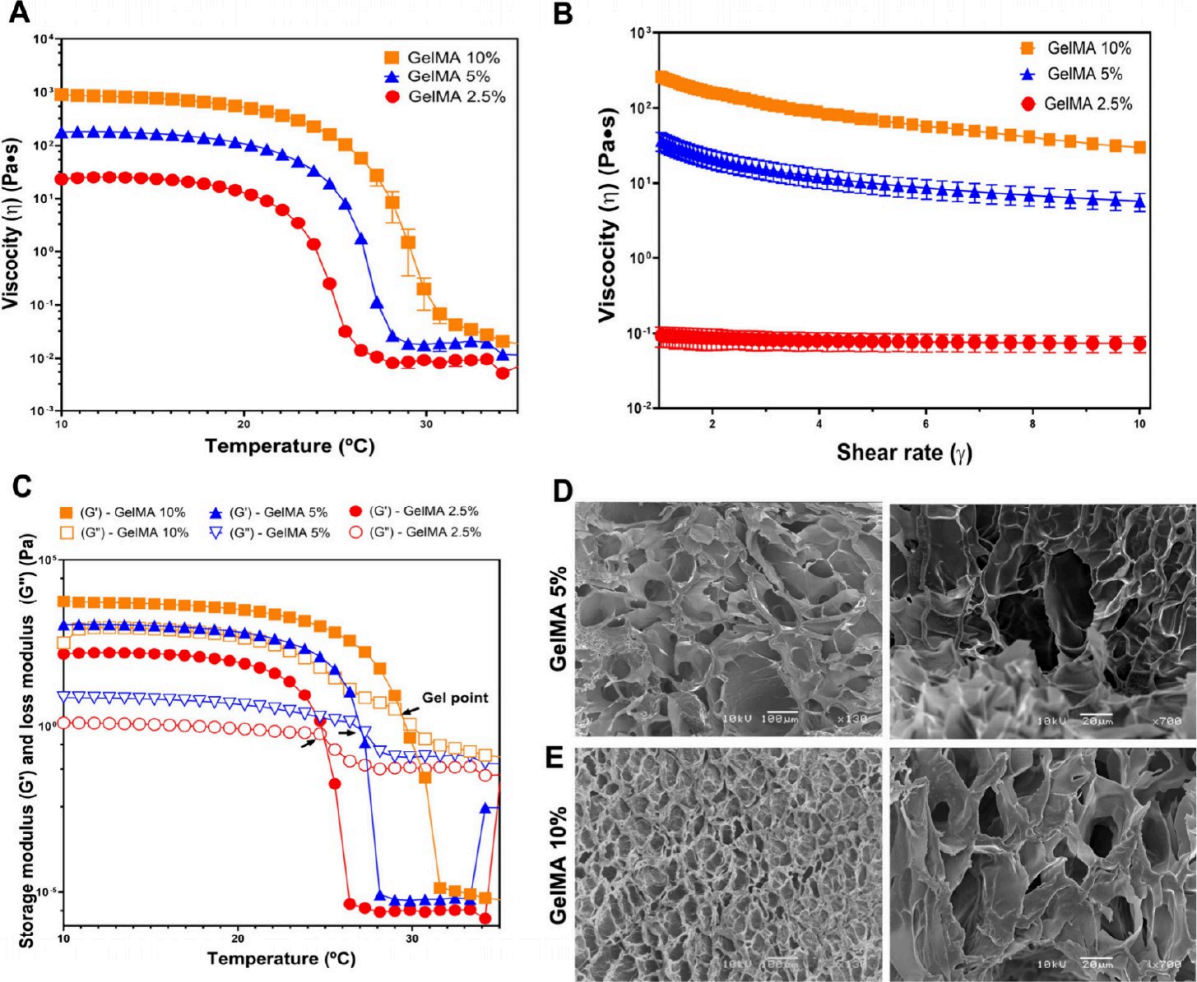
Rheological and ultrastructural characteristics of the hydrogel 2.5%, 5%, and 10% GelMA. (A) Viscosity as a function of the temperature. (B) Viscosity as a function of the shear rate at 22 °C. (C) Sweep temperature on *G*′ and *G*″. All analyses: *n* = 2. Black arrows indicate the gel points of each concentration. SEM characterization of GelMA constructs after UV photo-cross-linking of (D) 5% and (E) 10% (w/v) GelMA 24 h postlyophilization at 130× and 700× magnification, respectively. Scale bars: 20 and 100 μm. Values are represented as mean ± SEM. *p < 0.0332, **p < 0.0021, ***p < 0.0002, and ****p < 0.0001.

SEM was performed for 5% and 10% GelMA. SEM images show the presence of microporosity in both concentrations, indicating that, after photo-cross-linking, the porous network of GelMA is preserved, which is crucial for the release of biomolecules and fluids exchange. Evaluation of the cross section of the biomaterial revealed a tendency for larger pores in 5% GelMA (Figure 1D) compared to 10% GelMA (Figure 1E). Porosity analysis revealed that low-concentration GelMA (2.5%) exhibited a significantly higher porosity percentage compared to 5% (*p* < 0.0332) and 10% (*p* < 0.021). However, no significant difference was observed between the 5% and 10% GelMA groups (*p* > 0.05; Figure S2A,B). The pore area was higher in 2.5% GelMA compared to 10% GelMA (*p* < 0.0332). However, no significant difference was observed between the 5% and 10% GelMA groups (*p* > 0.05; Figure S2C).

### Viscoelastic Properties

The viscoelastic properties of GelMA (2.5%, 5%, and 10%) were evaluated after UV-light (365 nm) photopolymerization using an oscillatory rheometer (Figure 2A). These viscoelastic properties are crucial for assessing the biomaterial behavior postbiofabrication and its ability to maintain physical structures comparable to native tissue. All samples predominantly exhibited elastic behavior (*G*′ > *G*″) across all frequency ranges (Figure 2B). These findings suggest that GelMA after UV photopolymerization exhibits gel-like behavior with strong chemical bonds, determined by the absence of crossover between *G*′ and *G*″. Moreover, *G*′ was significantly higher in 10% GelMA, indicating enhanced mechanical properties and stability compared to 5% (*p* < 0.0001) and 2.5% (*p* < 0.0001) (Figure 2C). Figure 2D shows the damping factor tan δ = *G*′/*G*″ as a function of the frequency. The damping factor indicates the strength of a gel, with solid-like behavior dominating. Values of tan δ < 1 indicate strong gels, while values of tan δ > 1 indicate weaker gels. Therefore, at all frequencies evaluated, we observed strong gel behavior post-cross-linking.

**Figure 2:**
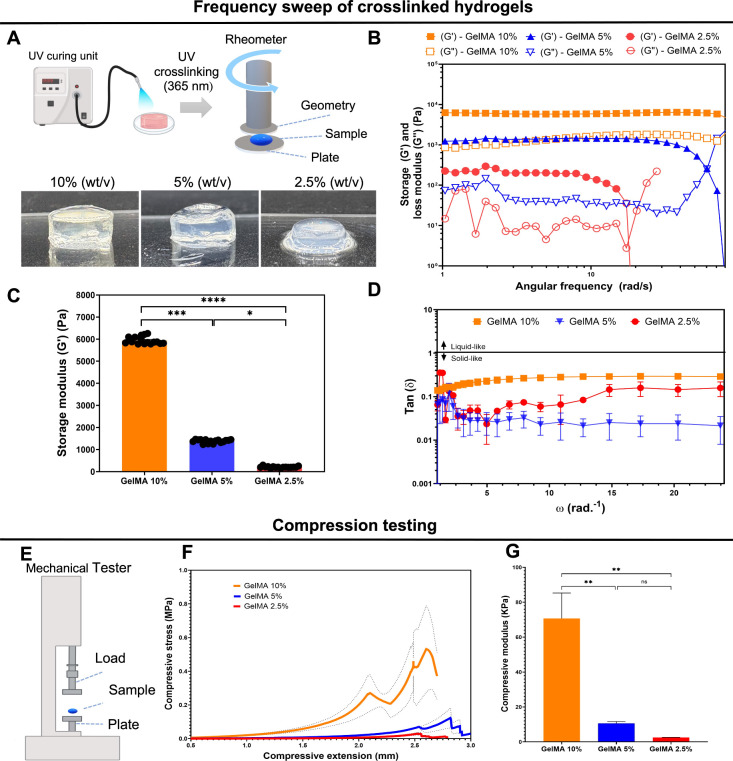
Frequency sweep analysis and compression testing of the hydrogel compositions. (A) Schematic diagram indicating how the samples were subjected to UV photocrosslinking (240 s, 18 mW cm^–2^) to obtain cross-linked 10%, 5%, and 2.5% GelMA hydrogels following of rotational rheometry. (B) *G*′ and *G*″ as a function of the angular frequency (ω). (C) Comparison of *G*′ between the groups. (D) Damping factor (tan δ) as a function of ω. All analyses: *n* = 2. (E) Compression test using a mechanical Instron. (F) Stress and extension curve from cross-linked hydrogels. (G) Compressive modulus mean values from cross-linked hydrogels (*n* = 3). Values are represented as mean ± SEM. *p < 0.0332, **p < 0.0021, ***p < 0.0002, and ****p < 0.0001.

Compression tests further assessed the mechanical properties of GelMA hydrogels (Figure 2E). Samples were evaluated after photocrosslinking (240 s, 365 nm, 18 mW cm^–2^) in a mechanical tester (round shapes of 7 mm diameter × 4 mm height) to obtain the stress–strain curve at a 1 mm min^–1^ compression rate (Figure 2F). The modulus was calculated in a strain segment of 0.05–0.15 mm mm^–1^. Compressive stress–strain curves demonstrated a positive correspondence to the GelMA concentration. 2.5% GelMA had 2.52 ± 0.17 kPa, 5% had 10.64 ± 1.74 kPa, and 10% had 70.72 ± 25.22 kPa compressive moduli (Figure 2G). Therefore, a significantly higher compressive modulus was observed in 10% GelMA compared to 5% (*p* < 0.0021) and 2.5% (*p* < 0.0021) GelMA (Figure 2G). Based on its superior mechanical properties, 10% GelMA was used for subsequent integration into NGCs and *in vivo* application.

### Bioactivity and Cytocompatibility

The amount of FGF-2 released from GelMA constructs was quantified daily and cumulatively over 30 days (Figure 3A). The daily release was increased in both GelMA concentrations (5% and 10%) during the first week, being 5% GelMA significantly higher on days 1, 3, 4, and 5 compared to 10% GelMA (*p* < 0.05; Figure 3B). Overall, there was substantial FGF-2 release in both concentrations during the first 7 days. After the first week, it decreased gradually and stabilized at 11 days, persisting for 30 days. After 11 days, approximately ∼1 ng mL^–1^ was released daily. The cumulative release shown in Figure 3C indicates a consistent tendency toward a gradual decrease of FGF-2 from the gel over time. The 5% and 10% GelMA formulations released 93.64 and 90.25 ng mL^–1^ after 30 days, respectively. The release rate was 12% from 5% GelMA compared to 11.58% from 10% GelMA. Significant amounts of FGF-2 remained in the constructs since 2 μg mL^–1^ was initially incorporated into the biomaterial.

**Figure 3:**
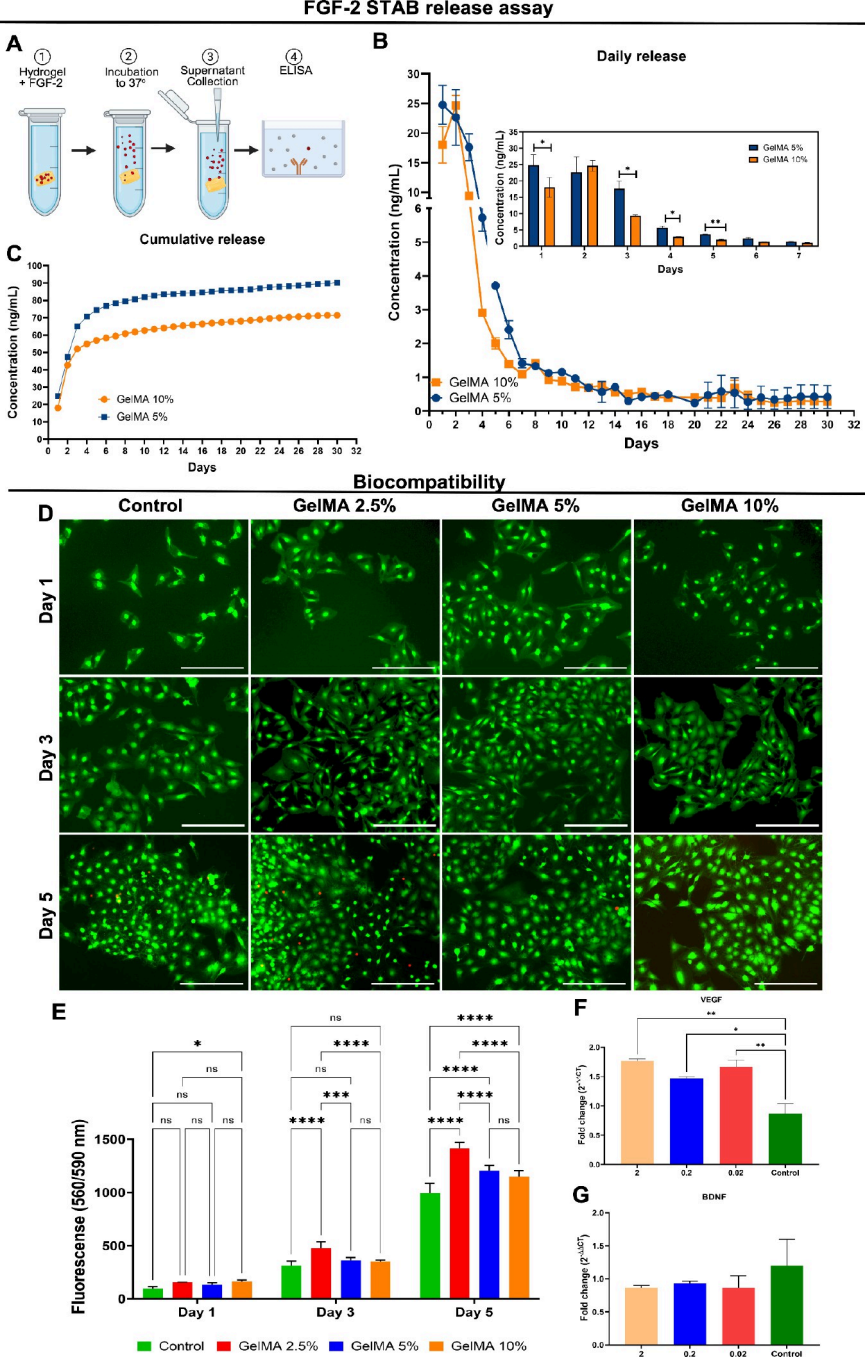
Quantification of FGF-2 release and cytotoxicity assessment in S16 cells. (A) Schematic diagram summarizing the FGF-2 release assay over 30 days. (B) Daily release evidenced a burst release in the first week. The inset in panel B compares the FGF-2 releases between 5% and 10% GelMA during the initial 7 days. (C) Cumulative release of FGF-2 over 30 days. (D) Representative images of S16 cells following indirect contact with GelMA+FGF-2, showing live cells (green, calcein AM) and dead cells (red, ethidium homodimer). (E) Presto Blue quantification in 1, 3, and 5 days following indirect exposure with GelMA+FGF-2. Scale bar: 300 μm. Relative gene expression of VEGF (F) and BDNF (G) after 3 days of stimulation of MSCs with FGF-2. All analyses: *n* = 3. Amplification of GAPDH was used as the endogenous gene. Data are presented as mean ± SEM. *p < 0.0332, **p < 0.0021, ***p < 0.0002, and ****p < 0.0001.

Indirect cytotoxicity was evaluated for 1, 3, and 5 days after indirect exposure to cross-linked GelMA hydrogels (2.5%, 5%, and 10%) containing FGF-2 (2 μg mL^–1^). Representative images from calcein AM staining (green) showed a high number of viable cells on days 1, 3, and 5, in all concentrations with very few dead cells (ethidium homodimer, red) on day 5 in control and GelMA 2.5% and 5% groups (Figure 3D). No statistically significant differences were observed in the percentage of cell viability among the experimental groups (*p* > 0.05; Figure S3). The cell viability and proliferation were further confirmed by Presto Blue assay (resazurin reduction; Figure 3E). On day 1, significant differences in metabolic activity were observed between the 10% GelMA group and the control (*p* < 0.0332). On day 3, significant increases in metabolism were seen in the 2.5% GelMA group compared to the control (*p* < 0.0001), 5% GelMA (*p* < 0.0002), and 10% GelMA (*p* < 0.0001). In comparison, no significant differences were observed between 5% and 10% GelMA compared to the control (*p* > 0.05). By day 5, all GelMA formulations (2.5%, 5%, and 10%) showed significant increases in metabolism compared to the control (*p* < 0.0001, respectively), with 2.5% GelMA demonstrating superior metabolic activity compared to both 5% (*p* < 0.0001) and 10% (*p* < 0.0001) (Figure 3E). These results suggest that exposure to GelMA hydrogels does not induce cytotoxicity and promotes increased metabolism and proliferation of Schwann cells, likely mediated by FGF-2 release *in vitro*.

Exploring direct encapsulation of cell-laden 5% and 10% GelMA hydrogels with human MSCs, we observed high viability in 5% GelMA between 70 and 83% and in 10% GelMA between 75 and 86% at 2, 7, and 15 days, respectively (Figure S4A). In addition, positive immunostaining for human mitochondria, as evidenced by punctate and filamentous structures within the cytoplasm, was observed at days 2, 7, and 15, indicating the presence of round-shaped cells with preserved metabolic activity (Figure S4B). No statistical differences in viability were observed in 5% GelMA (Figure S4C) and 10% GelMA (Figure S4D) (*p* > 0.05). A minor tendency to decrease in viability observed in 5% GelMA on day 15 and in 10% GelMA on day 2 could be attributed to UV-light exposure or effects related to the encapsulation process itself. These findings suggest that direct contact was safe.

To test the response of MSCs to FGF-2, we stimulated with 2, 0.2, or 0.02 μg mL^–1^ for 3 days. The gene expressions of VEGF and BDNF were evaluated in MSCs. Transcripts of VEGF were significantly increased following stimulation with FGF-2 compared to the unstimulated control at concentrations of 2 μg mL^–1^ (*p* < 0.05), 0.2 μg mL^–1^ (*p* < 0.05), and 0.02 μg mL^–1^ (*p* < 0.01) (Figure 3F). However, no statistical differences were detected between different concentrations (*p* > 0.05). These findings indicate upregulation of VEGF independent of the concentration of FGF-2 used. In contrast, transcripts of BDNF did not show statistically significant differences compared to the unstimulated control at concentrations of 2, 0.2, and 0.02 μg mL^–1^ FGF-2 (*p* < 0.05) (Figure 3G).

### Biofabrication of Functionalized NGCs

To analyze the 3D printing resolution and capacity, bidimensional PCL scaffolds were examined using SEM images for the thickness, pore dimensions, and filament diameter (Figure S5A–E). The parameters consisted of a 0.4 mm stainless steel nozzle, a pressure range of 180–200 KPa, a thermoplastic head temperature between 180 and 200 °C, a bed temperature of 10 °C, and a printing speed of 7–10 mm s^–1^. The qualitative analysis revealed a smooth architecture, round-shaped filaments, and no fusion between layers, demonstrating excellent morphology and dimensions. Quantitative findings revealed adequate dimensions and high printability (*Pr* = 1.02; Figure S5E). These results indicate the excellent 3D printing performance of PCL polymers, demonstrating high resolution, well-formed filaments, and a suitable microstructure for the subsequent 3D printing of NGCs.

During NGCs fabrication, the external wall of the NGCs was manufactured by continuous spiral deposition of PCL. SEM revealed a tubular structure with uniform filaments and a smooth surface. The transverse section on the dorsal view presented round-shaped filaments, preserving circularity and reproducibility (Figure 4A–C). In the top view of the cross section, the circularity of the tubular structure with regularity in the inner diameter and wall was observed (Figure 4D,E). The constructs exhibit an inner diameter (ID) of 2106 ± 29.31 μm and an external diameter (ED) of 2488 ± 34.73 μm with a wall thickness of 382.3 ± 8.41 μm (Figure 4F).

**Figure 4:**
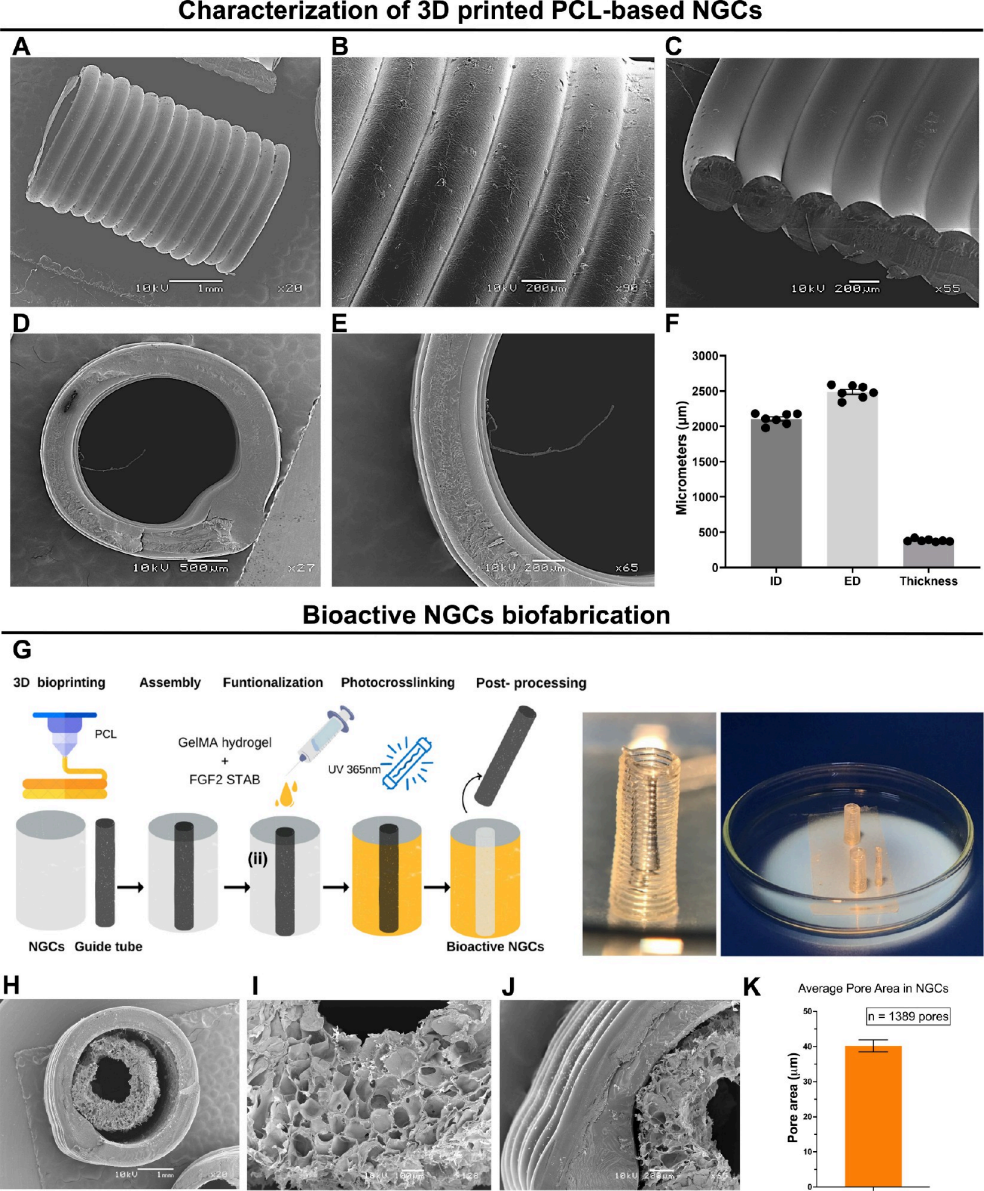
Structural characterization and fabrication process of 3D-printed NGCs. (A) Top, (B) dorsal, and (C) fractured lateral views of the 3D-printed NGCs featuring a spiral pattern and distinct individual filament structures. Cross-sectional views at midlevel of the NGCs at low (D) and high (E) magnification. (F) Analysis of the dimensions of NGCs including the inner diameter, external diameter, and wall thickness. Scale bars: 1 mm and 200 and 500 μm. (G) Schematic diagram illustrating the procedures involved in the biofabrication of NGCs and their macroscopic appearance. (H) Top view of the NGCs following cross-linking of the internal 10% GelMA layer incorporated with FGF-2. (I) Detailed view showing preservation of the NGC lumen after removal of the guide tube. (J) Magnified view of the interface between PCL and GelMA. (K) Analysis of the average pore size on the internal wall of 10% GelMA based on SEM images (*n* = 3). Scale bars: 200 μm and 1 mm. Data are presented as mean ± SEM.

The 3D printing process of a mechanically stable NGC was performed in a hybrid system from PCL and 10% GelMA. Initially, 3D printing of NGCs (3 mm × 10 mm) and guide tubes (2 mm × 10 mm) was developed using PCL (Figure 4G). The guide tube was then inserted and centered in the NGCs. Finally, 10% GelMA containing FGF-2 (2 μg mL^–1^) was injected into the space between the outer wall and the guide tube and photocrosslinked with UV light (365 nm) (Figure 4G). A hybrid tubular structure composed of two concentric cylinders of PCL was then prepared (Figure 4H). The constructs filled with GelMA+FGF2 hydrogel were then obtained, preserving the lumen of the tubular structure (Figure 4I). SEM images showed that GelMA remained adhered to the internal wall of the construct, maintaining the tubular lumen and external and internal microporosity (Figure 4J). The average pore size of 10% GelMA within the inner wall of the NGCs was 40.22 ± 1.70 μm, based on the analysis of 1389 pores, and was comparable to that of bulk 10% GelMA hydrogels (48.48 ± 6.13 μm) (Figure 4K).

For the characterization of *in vitro* enzymatic degradation and swelling, UV-photocrosslinked GelMA hydrogels at 10% (w/v), used for NGC fabrication, were compared with lower concentrations of 2.5% and 5% (w/v). The 10% GelMA hydrogels exhibited slower degradation (6 days) compared to 5% GelMA (5 days) and 2.5% GelMA (4 days), indicating that higher GelMA concentrations reduce enzymatic degradation (Figure S6A). The swelling ratios of GelMA hydrogels at different concentrations are shown in Figure S6B. After 24 h, the swelling ratios reached 8.81 ± 1.04 for 2.5% GelMA, 3.90 ± 0.47 for 5% GelMA, and 2.40 ± 0.21 for 10% GelMA, indicating that increased concentration and cross-linking reduce water absorption.

### PNI and Repair: *In Vivo* Study

Long-gap PNI results in degeneration of the distal nerve stump, characterized by axonal and myelin loss as well as muscle atrophy. Delayed intervention further compromises the regenerative potential of the distal stump, leading to reduced axonal regrowth, impaired remyelination, and limited functional recovery (Figure 5A). The biofabrication of NGCs with controlled growth factor release to enhance nerve regeneration represents a promising translational approach (Figure 5B). In our study, the rat PNI model (8 mm critical gap) was implemented to evaluate the performance of NGCs developed above (Figure 5B). Microsurgical approaches consisted of an autograft to simulate standard of care (SoC) surgery. In addition, biofabricated NGCs were implanted with or without FGF-2 (Figure 5C). Implantation of NGCs after sciatic nerve transection in rats was reproducible and relatively rapid. Epineural suture application showed low complexity due to the transparency of the conduit. Additionally, the dimensions of the conduit were suitable for the nerve size and gap (Figure 5C). No wound dehiscence or signs of inflammation were observed after the implantation of NGCs in the experimental animals at 12 weeks postsurgery.

**Figure 5:**
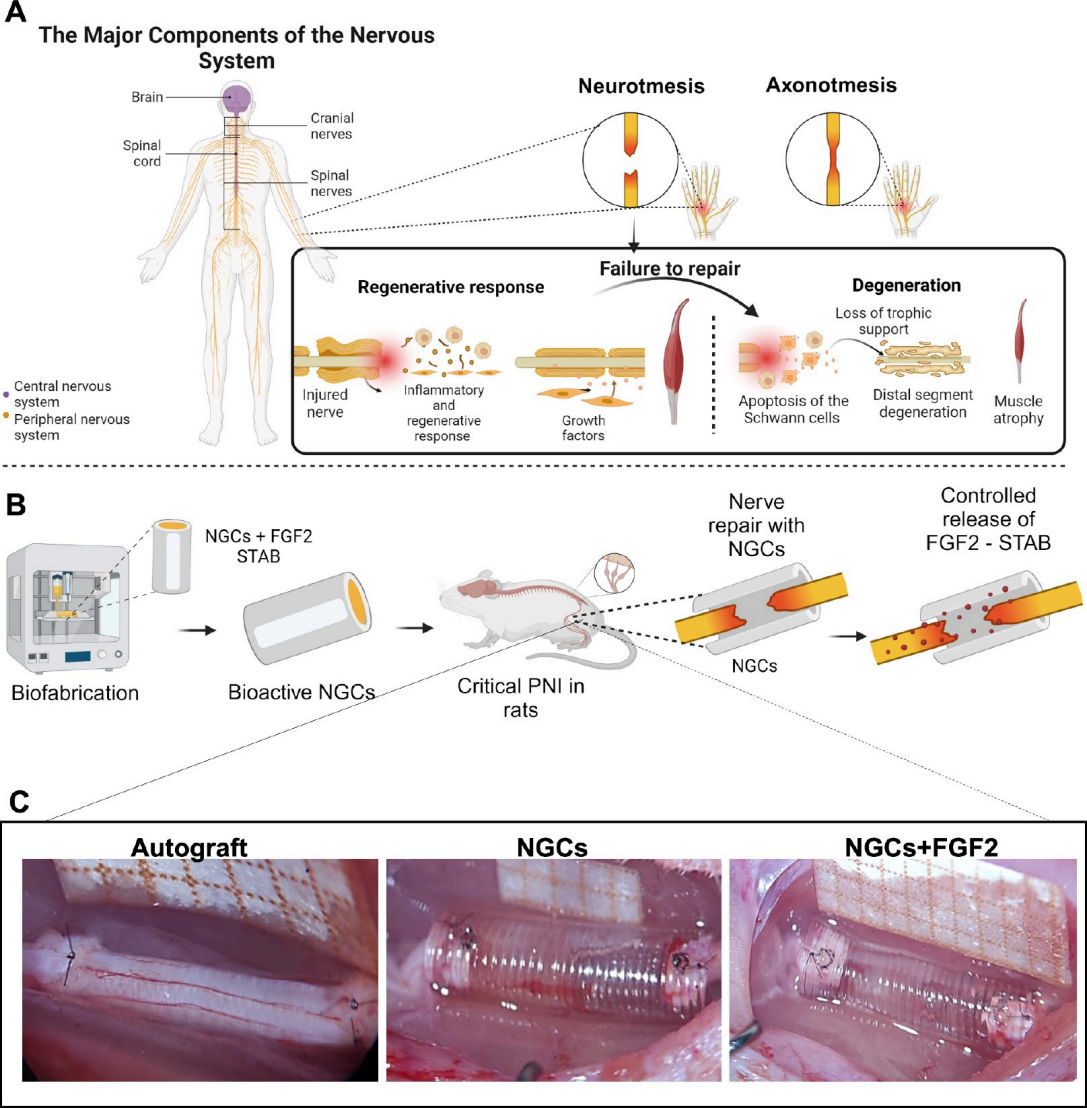
Experimental sciatic nerve injury in the autograft, NCGs, and NGCs+FGF2 groups. (A) Schematic diagram demonstrating how the regenerative process is irreversible after long-gap PNI lesions. (B) Bioactive NGCs fabricated by using 3D printing and implanted into an 8-mm long-gap PNI rat model for subsequent functional and morphological evaluation. (C) In the autograft group, sciatic nerve neurotmesis (8 mm critical defect) performed, repositioned, and repaired with epineurial sutures. In the NGCs and NGCs+FGF2 groups, critical defect of the sciatic nerve was created, and then the nerve stumps were inserted 1 mm into the NGCs and fixed by epineural sutures. Scale bar: 10 mm.

### Motor, Sensorial Functional, and Electrophysiological Recovery Evaluation

A catwalk platform and an electronic von Frey aesthesiometer were utilized to evaluate motor and sensory recovery after NGCs implantation in experimental groups (Figure 6A). During the evaluation of the peroneal nerve functional index between 2 and 7 weeks, no significant differences were detected among the autograft, NGCs, and NGCs+FGF2 groups (*p* > 0.05). Nonetheless, significant differences were observed at 11 and 12 weeks between the autograft and NGCs+FGF2 groups compared to the NGC group (*p* < 0.0332 and 0.0021), respectively. Additionally, no significant differences between the autograft and NGCs+FGF2 groups were observed at 11 and 12 weeks (Figure 6B). These findings indicate improved functional motor recovery in animals treated with autografts and NGCs+FGF2. Moreover, the degree of functional recovery was similar between the autograft- and NGCs+FGF2-treated groups.

**Figure 6:**
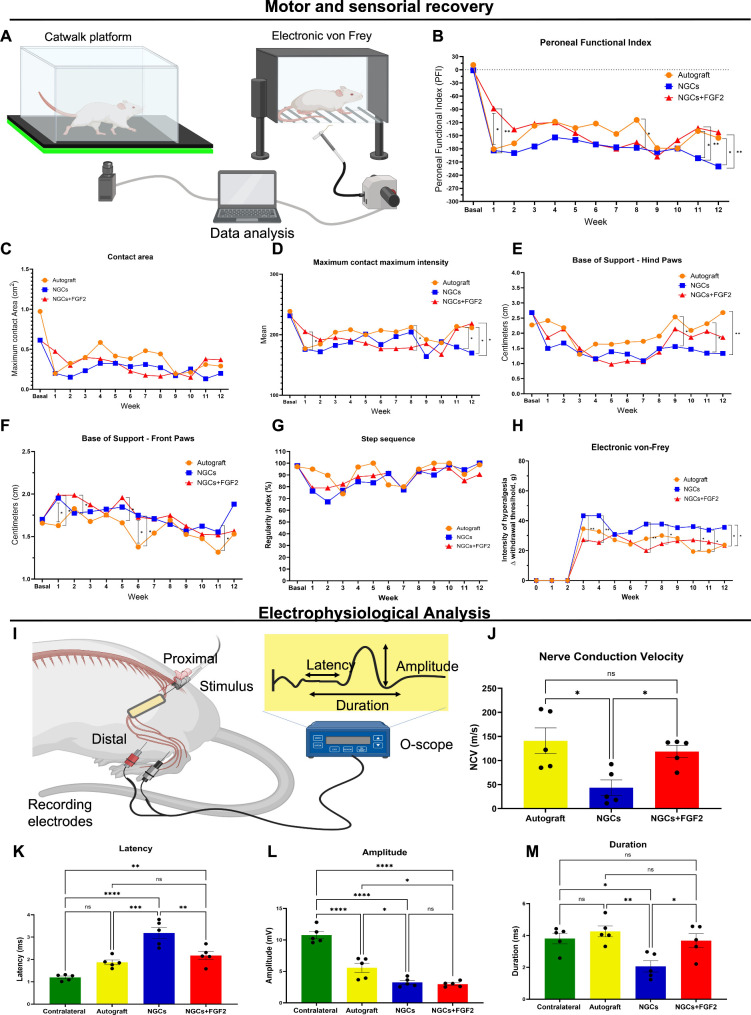
Analysis of the locomotor, sensorial, and electrophysiological recovery for 12 weeks after nerve repair. (A) Schematic illustrations of the catwalk platform system and the electronic von Frey system. Comparisons of the peroneal functional index (B), contact area (C), maximum contact area (D), base of support for hindlimbs (E) and forelimbs (F), regularity index (G), and withdrawal threshold (Δ withdrawal thresholds) (H) across the experimental groups. (I) Electrophysiological procedure to obtain an CMAP from the tibialis cranialis muscle. (J) Nerve conduction velocity obtained after proximal and distal stimulation. Measurements included (K) latency, (L) amplitude, and (M) duration in the experimental groups at 12 weeks. All analyses: *n* = 5. The values are represented as mean ± SEM. *p < 0.0332, **p < 0.0021, ***p < 0.0002, and ****p < 0.0001.

During the analysis of the contact area, no significant differences were observed between the groups (*p* > 0.05). However, the maximum contact area showed significant differences between the autograft and NGC+FGF2 compared to the NGC group (*p* < 0.0332) in weeks 11 and 12 (Figure 6C,D). This indicates an increase in the contact surface area of the injured limb in both the autograft and NGCs+FGF2.

During analysis of the base of support for the hindlimbs, significant differences were observed at 9, 11, and 12 weeks between the autograft and NGCs counterparts (*p* < 0.0332 and 0.0021, respectively). However, no differences were observed between the NCGs and NGCs+FGF2 groups (*p* > 0.05) (Figure 6E). An increase in the injured limb support in the autograft-treated group and a trend toward increased support in the NGCs+FGF2 group were observed. In contrast, the NGCs showed a low support for the injured limb. On the other hand, during analysis of the base support for the forelimb, there was a trend toward decreased support of thoracic limbs in all experimental groups from week 7, indicating a shift in weight-bearing toward the pelvic limbs (Figure 6F).

Regarding gait regularity, no significant differences were observed between the experimental groups (*p* > 0.05) (Figure 6G) at all evaluated time points. These findings indicate consistent gait patterns during locomotion across the groups.

The hyperalgesia after mechanical stimulation showed differences between the autograft and NGCs at weeks 10, 11, and 12 (*p* < 0.05). Significant differences were also observed between the NGCs+FGF2 and NGCs groups at weeks 3, 4, and 7 (*p* < 0.0021), respectively. Besides, there were significant differences at weeks 8 and 12 when the NGCs+FGF2 and NGCs groups were compared (*p* < 0.0332) (Figure 6). Overall, the present findings indicate that the autograft and NGCs+FGF2 groups showed reduced hyperalgesia intensity, potentially controlling neuropathic pain over 12 weeks, with both groups being equivalent (Figure 6H).

Electrophysiological analysis was performed at 12 weeks in rats under general anesthesia by direct stimulation of the sciatic nerve proximal to the lesion site. Active electrodes were placed in the tibial cranial muscle to obtain CMAP (Figure 6I). Significant differences were observed in the nerve conduction velocity (NCV) at 12 weeks between the autograft and NGCs+FGF2 groups compared to the NGCs group (*p* < 0.0332) (Figure 6J). The evaluation of latency showed that the autograft group presented superior results without differences compared to the contralateral side (*p* > 0.05). Besides, no statistical differences between the autograft and NGCs+FGF2 (*p* > 0.05) were observed. Therefore, latency was significantly decreased over time in the autograft (*p* < 0.0021) and NGCs+FGF2 (*p* < 0.0002) compared to NGCs (Figure 6K).

Regarding the amplitude, the autograft, NGCs, and NGCs+FGF2 were significantly reduced compared to the contralateral side. Autograft groups showed greater amplitudes compared to the NGCs and NGCs+FGF2 groups (*p* < 0.05) (Figure 6L). Finally, the CMAP duration was nonsignificant between the autograft and NGCs+FGF-2 (*p* > 0.05) and was longer in the contralateral (*p* < 0.032), autograft (*p* < 0.0021), and NGCs+FGF2 (*p* < 0.032) compared to NGCs group (Figure 6M).

### Muscle and Corporal Mass

The macroscopic appearance of muscle specimens evidenced a tendency toward muscle mass recovery, being more evident in gastrocnemius muscles in the autograft and NGCs+FGF2 compared to contralateral muscles (Figure 7A). Significant differences were observed between the contralateral tibialis cranialis and gastrocnemius muscles compared to the autograft, NCGs, and NGCs+FGF2 groups, respectively (*p* < 0.0001). When the autograft was compared with the NGCs and NGCs+FGF2 groups, significant differences were observed at week 12 in the tibialis cranialis muscle (*p* < 0.01) and gastrocnemius muscle (*p* < 0.0001), respectively (Figure 7B,C). However, no significant differences were observed between the NGCs and NGCs + FGF2 groups in both tibialis cranialis and gastrocnemius muscles (*p* > 0.05) (Figure 7B,C). These findings correlate with an increased neuromuscular reinnervation in the autograft-treated group. Regarding body mass, no significant differences were observed over 12 weeks among the groups (*p* > 0.05) (Figure 7D). The findings indicate a normal linear growth curve.

**Figure 7:**
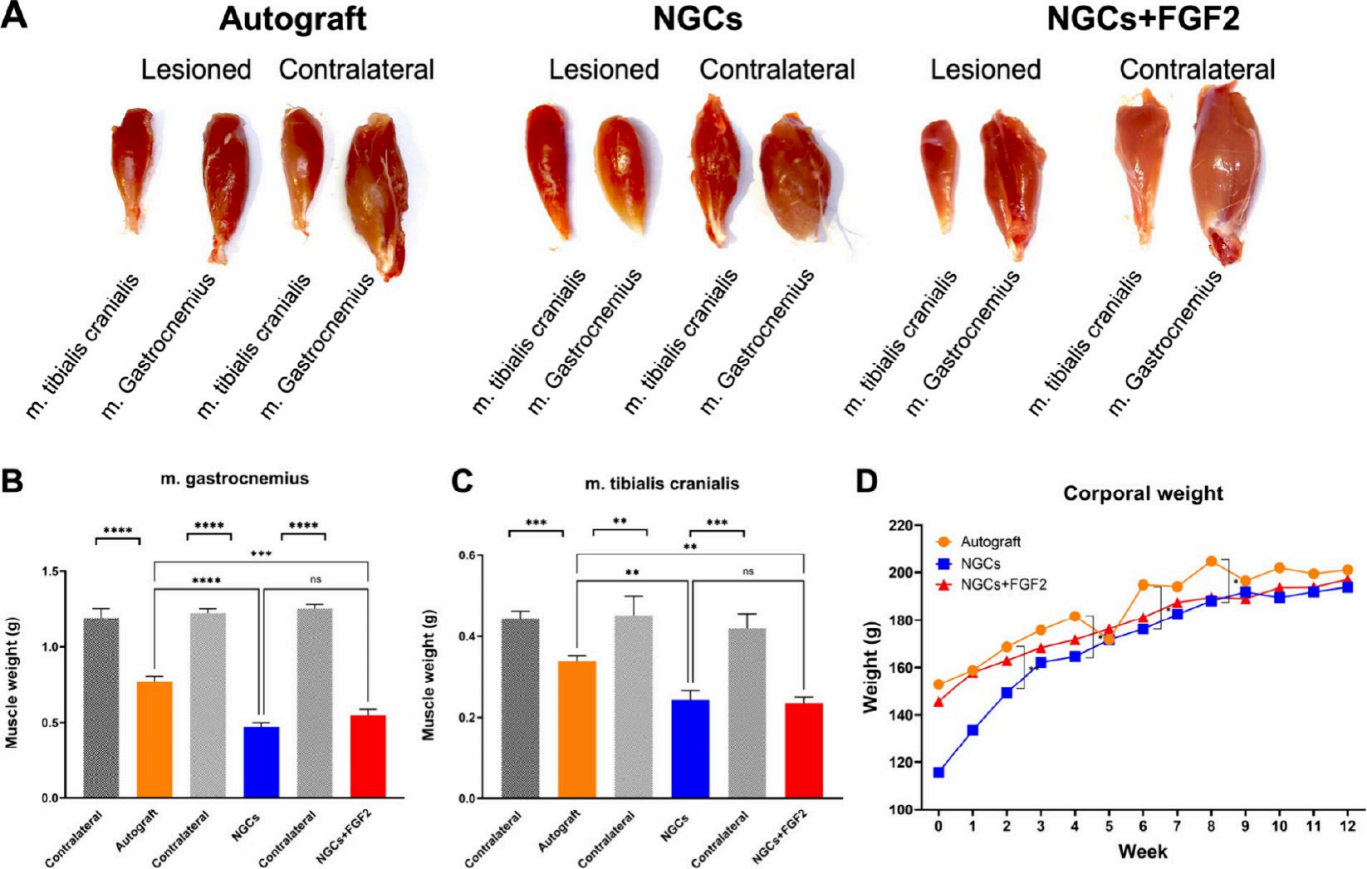
Analysis of neuromuscular reinnervation 12 weeks postnerve repair. (A) Microscopic comparison of the lesioned and contralateral tibialis cranialis and gastrocnemius muscles across the experimental groups. Graphs show comparative data for gastrocnemius muscle weight (B), tibialis cranialis muscle weight (C), and overall body weight (D) among the autograft, NGCs, and NGCs+FGF2 groups. All analyses: *n* = 5. The values are represented as mean ± SEM. *p < 0.0332, **p < 0.0021, ***p < 0.0002, and ****p < 0.0001.

### Immunohistochemistry

Representative images showed increased S100 immunostaining in all of the experimental groups (Figure 8A). Among these, a significant increase was observed in the NGCs+FGF2 group compared to the autograft (*p* < 0.0332) and contralateral nerve (*p* < 0.0001) (Figure 8B). No significant differences were found between the NGCs and NGCs+FGF2 groups (*p* > 0.05). These findings suggest enhanced Schwann cell reactivity, which was more prominent in the group treated with NGCs containing the controlled release of FGF2.

**Figure 8:**
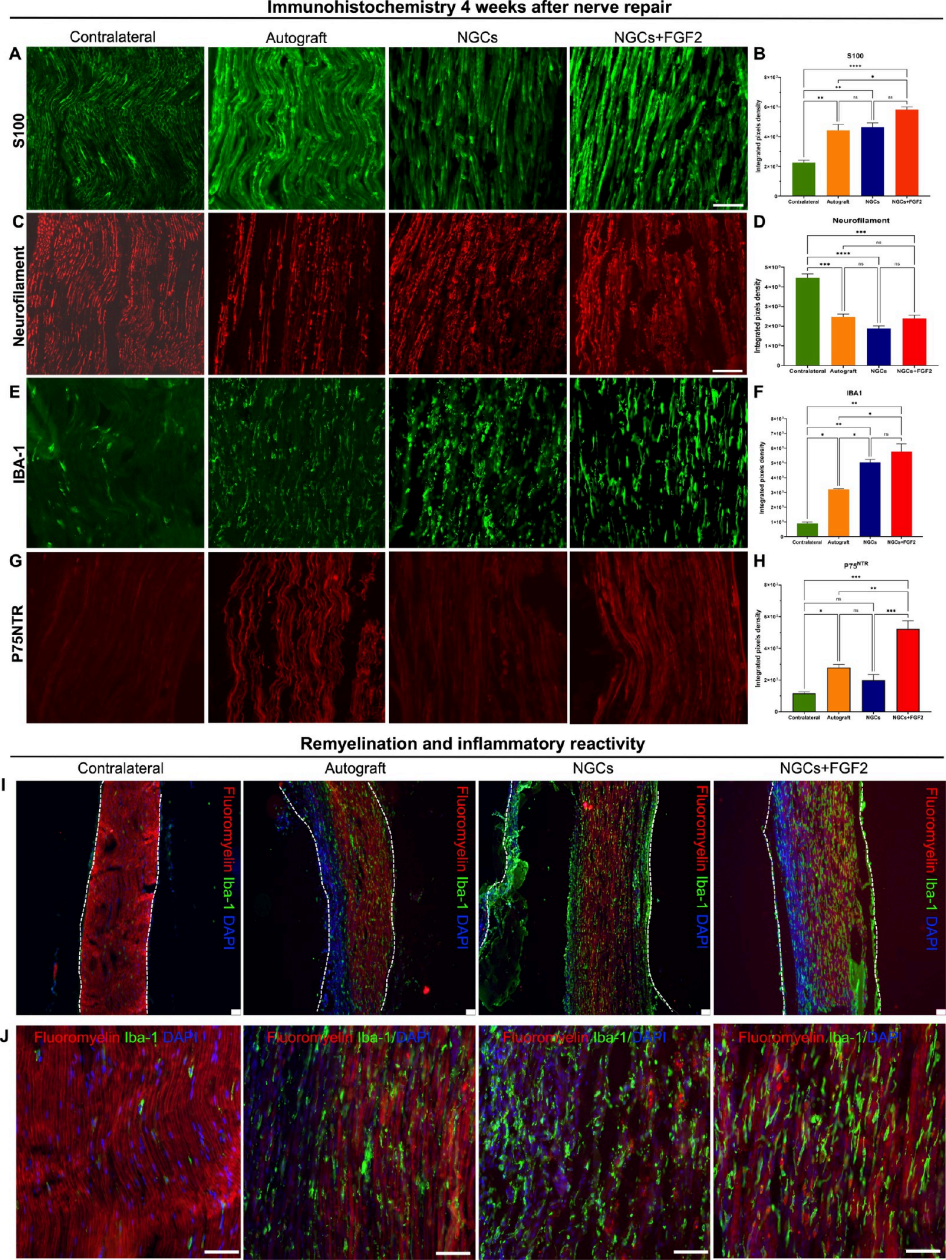
Analysis of immunostaining for cytoskeletal components, neurotrophin receptors, degree of myelination, and inflammatory reactivity 30 days postinjury in autografts, NGCs, and NGCs with FGF-2 groups. Immunostaining included S100 (A), neurofilament (C), IBA-1 (E), and P75NTR (G), with corresponding quantification of the integrated pixel density shown in parts B, D, F, and H, respectively. Representative low-magnification images (I) at 10× demonstrate myelin sheath labeling with fluoromyelin (red) and macrophages with IBA-1 (green), indicating reinnervation of proximal and distal nerve stumps. High-magnification images (J) at 40× show triple labeling with IBA-1 (green), DAPI (blue), and fluoroyelin (red). All analyses: *n* = 3. The values are represented as mean ± SEM. *p < 0.0332, **p < 0.0021, ***p < 0.0002, and ****p < 0.0001.

During the evaluation of cytoskeletal organization through neurofilament protein, significant differences were observed when the contralateral nerves across the autograft, NGCs, and NGCs+FGF2 groups were compared (Figure 8C). Among the experimental groups, there were no significant differences between the autograft, NGCs, and NGCs+FGF2 groups (*p* > 0.05) (Figure 8D). These findings show an increase in cytoskeletal organization after injury and repair in all experimental groups, with more pronounced effects observed in the autograft and NGCs+FGF2 groups.

Representative images showed an increase in IBA-1 immunostaining in all experimental groups, which was more notable in the autograft and NGCs-FGF2 groups (Figure 8E). The macrophage reactivity through immunostaining of IBA-1 protein evidenced a significant increase compared to the contralateral nerve across the autograft, NGCs, and NGCs+FGF2 groups. Among the experimental groups, a significant increase in IBA-1 was observed in the NGCs and NGCs+FGF2 groups compared to the autograft (*p* < 0.0332). However, there were no significant differences between the NGCs and NGCs+FGF2 groups (*p* > 0.05) (Figure 8F). These findings suggest that GelMA possibly increases the macrophage activity. During the evaluation of receptors P75NTR, representative images showed an increase in P75NTR immunostaining and superior organization, which is more evident in the autograft, NGCs, and NGCs+FGF2 groups (Figure 8G). Significant expression was observed in the NGCs+FGF-2 group compared to the contralateral (*p* < 0.0002), autograft (*p* < 0.0021), and NGCs (*p* < 0.0021) groups (Figure 8H). These findings suggest that our approach is neuroprotective, as indicated by the upregulated expression of neurotrophin receptors, potentially localized to Schwann cells or within the Bands of Büngner.

Myelinated axon recovery was evaluated by fluoromyelin staining, which revealed continuous myelin sheath formation and complete reconnection of the nerve stumps in the autograft, NGCs, and NGCs+FGF2 groups (Figure 8I). The autograft and NGCs+FGF2 groups exhibited higher red fluorescence, indicating greater presence and organization of myelinated axons than the NGCs group. Additionally, in the NGCs+FGF2 group, an increase in nuclei labeling with DAPI was noted, indicating greater cell proliferation (Figure 8J).

### Ultrastructural and Morphological Analysis

The ultrastructural evaluation by TEM revealed an improvement in the number and organization of nerve fibers observed in the autograft and NGCs+FGF2 groups compared with NGCs (Figure 9A). Despite axons in the NGCs+FGF2 group being smaller in diameter, there was an increase in their number compared to the autograft and NGCs. The diameter of large axons surrounded by myelin sheath showed a larger diameter and similar thickness compared to the contralateral side in the autograft and NGCs+FGF2 groups compared to the NGCs group (Figure 9A). Collagen fibers within the endoneurium, consisting of thin cylindrical lamina, were found to be distributed around the motor axons. A greater quantity of collagen fibers was noted in the NGCs-treated groups compared to the autograft group (Figure 9A). In addition, hematoxylin and eosin (H&E) staining of the midsections inside the NGCs, with the PCL layer removed, revealed that the autograft group exhibited thicker nerves with more connective perineural tissue and the presence of aligned, well-organized fibers. The NGCs groups showed disorganized and sparse nerve fiber arrangements growing adjacent to the persistent GelMA internal layer. In contrast, the NGC+FGF2 group demonstrated organization, forming parallel bundles that were well distributed and aligned along the persistent GelMA internal wall (Figure S7).

**Figure 9:**
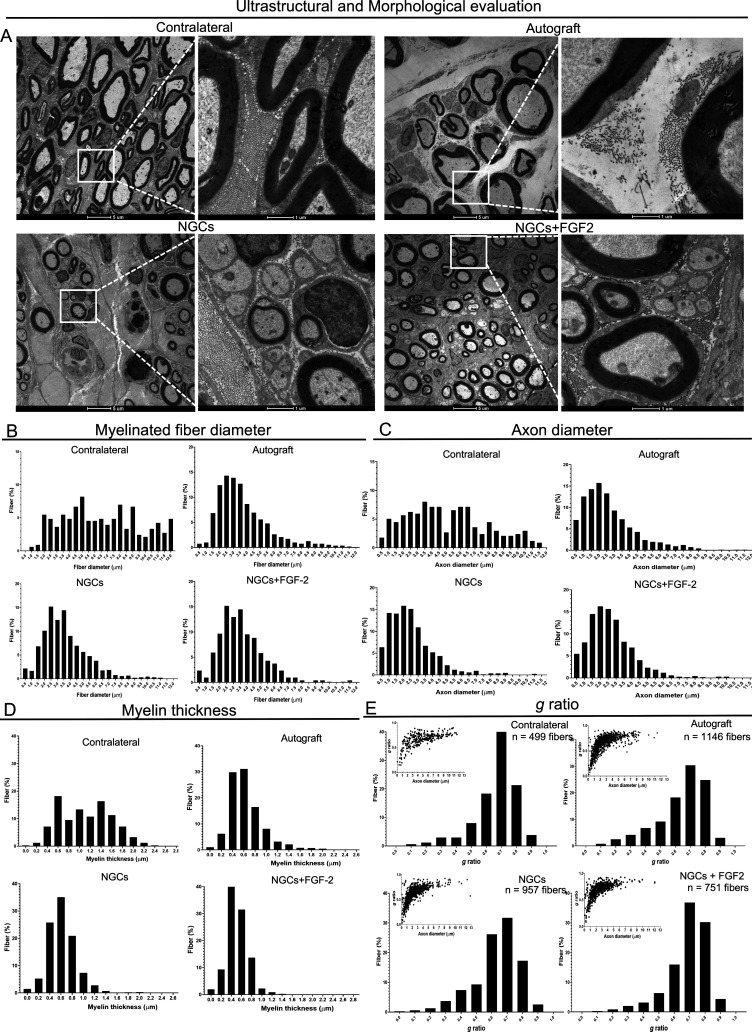
Ultrastructural and morphometric analysis of the myelinated fibers, axon diameters, myelin sheath thicknesses, and *g* ratios was performed at 12 weeks following PNI and repair in the experimental groups. (A) High-resolution TEM images showing the organization and distribution of myelinated axons, myelin sheath thicknesses, and endoneural collagens across the groups at 12 weeks. Measurements of the fiber diameter (B), axon diameter (C), myelin sheath thickness (D), and *g* ratio (E) were obtained from motor axons and compared among the contralateral, autograft, NGCs, and NGCs + FGF2 groups. The insets in part E present the correlation analysis between the *g* ratio and axon diameter (μm). All analyses: *n* = 5. Scale bars: 1 and 5 μm.

Morphometric analysis of the fiber diameter (axon plus myelin sheath) 12 weeks postsurgery revealed the following distributions: contralateral nerves ranged from 5 to 8.5 μm (mean 7.05 ± 2.11 μm), autograft from 2 to 4 μm (mean 3.7 ± 1.89 μm), NGCs from 2 to 3.5 μm (mean 3.5 ± 1.75 μm), and NGCs+FGF2 from 2.5 to 4.5 μm (mean 3.82 ± 1.91 μm). (Figure 9B).

Morphometric analysis of axon diameters at 12 weeks postsurgery showed the following ranges and mean values: contralateral nerves ranged from 2 to 8 m (mean 5.8 ± 2.4 μm), autograft from 1 to 3 μm (mean 2.6 ± 1.7 μm), NGCs from 0.5 to 3 μm (mean 2.5 ± 1.54 μm), and NGCs+FGF2 from 1 to 3.5 μm (mean 2.8 ± 1.76 μm) (Figure 9C).

Morphometric analysis of the myelin thickness in regenerated nerves at 12 weeks postsurgery revealed the following values: contralateral nerves ranged from 1.2 to 1.6 μm (mean 1.48 ± 0.91 μm), autograft from 0.3 to 1.0 μm (mean 0.63 ± 0.32 μm), NGCs from 0.4 to 0.8 μm (mean 0.62 ± 0.27 μm), and NGCs+FGF2 from 0.4 to 0.8 μm (mean 0.63 ± 0.28 μm) (Figure 9D).

Measurements of the *g* ratio at 12 weeks are as follows: contralateral (0.6–0.8 μm, mean 0.72 ± 0.47), autograft (0.6–0.8 μm, mean 0.63 ± 0.16), NGCs (0.6–0.80 μm, mean 0.62 ± 0.14), and NGCs+FGF2 (0.64–0.14 μm, mean 0.63 ± 0.01) (Figure 9E). Correlation analysis between the *g* ratio and axon diameter revealed a shift toward thinner fibers in the experimental groups compared to the contralateral nerves. However, a comparable distribution pattern was observed in the autograft and NGCs+FGF2 groups at 12 weeks, as illustrated in the insets onto Figure 9E.

## Discussion

Long-gap and delayed PNI repairs remain a clinical challenge due to insufficient neurotrophic support and an unsustained distal proregenerative environment, leading to poor axonal regeneration and low recovery rates.^5,9^ Commercial hollow NGCs have shown limited short-gap repair success due to their acellular nature, lack of trophic support, poor Schwann cell substrate, and restricted geometric customization.^6,21,29^ Autografts are the most effective surgical strategy, although limited by donor nerve availability, length, and caliber mismatches, which can result in poor restoration and sensorimotor function.^6^ To overcome these limitations, we developed an innovative biofunctional NGC for nerve regeneration, featuring a support-free, self-buildable architecture, and personalized design via 3D microextrusion printing. The NGCs were functionalized with a highly porous, biomimetic GelMA hydrogel surface to support axon regeneration and Schwann cell infiltration and alignment. Additionally, we controlled the release of a genetically engineered variant of FGF-2 with thermostability, which enhanced Schwann cell proliferation, myelination, axonal regrowth, and functional recovery in a long-gap PNI model in rats.

The mechanical and biological properties of GelMA-based hydrogels are widely dependent on the DoF, polymer concentrations, photoinitiator, and thermal gelation as well as UV exposure (intensity and time) during polymer photoreticulation.^33^ Ninhydrin assay was an accurate, practical, and cost-effective strategy for determining the DoF.^34^ We synthesized GelMA samples with a DoF of 74.06–74.28%. An estimated DoF of 50% is the minimum required to generate GelMA-based hydrogels at 5% (w/v). Our findings are comparable to previous studies that achieved a high DoF (∼75–80%).^34,35,40^ These findings were confirmed through enhanced mechanical properties in photocrosslinking assay and rheology parameters. Photocrosslinkable biomaterials such as GelMA enable the construction of various 3D architectures while preserving cellular viability.^40–42^ GelMA samples exhibited rapid cross-linking, forming constructs with good mechanical properties. Using 0.5% irgacure 2959 concentration and 10% GelMA, stable hydrogel was created after exposure to UV light (365 nm, 18 mW cm^–2^) for 200 s. These findings allowed the optimization of cross-linking parameters to cast a tubular structure from GelMA into the PCL NGCs.

During rheological evaluation of the hydrogel, a pseudoplastic (shear-thinning) behavior was observed at all concentrations, with the 10% GelMA formulation exhibiting the most pronounced effect at 22 °C. Temperature ramp analysis further demonstrated pseudoplastic and gel-like behavior in the 10% GelMA formulation, particularly during cooling, compared to the 2.5% and 5% concentrations. Additionally, thermostability was observed at room temperature between 20 and 28 °C in the 5% and 10% GelMA samples, as previously observed.^36^ Although the rheological results showed good viscosity and *G*′ in 2.5% and 5% GelMA, the results were not superior to those in 10% GelMA.

Viscoelastic properties are crucial for understanding the biomaterial’s ability to maintain physical structures like native tissue.^36^ We analyzed cross-linked samples and observed a predominance of elastic properties, with *G*′ superior to *G*″ at 37 °C. After photopolymerization with UV light, all GelMA concentrations exhibit a gel-like behavior. The *G*′ modulus was increased in 10% GelMA samples with more stability during increasing frequency. The damping factor indicates a dominant solid-like behavior (tan δ < 1) across all GelMA concentrations. In this context, a highly porous surface was observed in GelMA both before and after its incorporation into the NGCs. Therefore, 10% GelMA revealed thermostability before cross-linking and improved stability after cross-linking, as well as high porosity. These parameters allowed 10% GelMA hydrogel to be incorporated into the inner wall of NGCs with the aim of contacting the native nerve during the regeneration process.

The biofabrication of biomaterial-based constructs requires high cytocompatibility to maintain the healing mechanism of endogenous tissue cells.^33,38^ We observed high viability of Schwann cells (S16 cells) with an exponential increase in metabolism and proliferation by day 5 across all GelMA concentrations (2.5%, 5%, and 10%), with the most pronounced effect seen in the 2.5% GelMA group. Therefore, in addition to the absence of toxicity in Schwann cells, our results confirm that FGF-2 released from the GelMA hydrogels can enhance the proliferation and metabolism of endogenous cells over extended periods.^12,43^ These findings are consistent with previous studies demonstrating increased Schwann cell proliferation, promotion of nerve regeneration mediated by FGF-2 *in vivo*, and the absence of toxicity in Schwann cells encapsulated in GelMA hydrogels for 3D bioprinting applications.^14,43,44^ In addition, high viability of human MSCs after 15 days of encapsulation in GelMA was observed. Our results were in line with previous studies showing a high cell viability (>95%) of MSCs in the first 7 days after encapsulation.^38,45^ Previously, three regimes of UV-light intensity and exposure time on human MSCs were analyzed under similar conditions (10% GelMA + 0.5% irgacure 2959): 700 mW cm^–2^ for 37 s (high), 100 mW cm^–2^ for 100 s (medium), and 10 mW cm^–2^ for 316 s (low). Similar to our results using 18 mW cm^–2^ for 240 s (low), the study showed 91% viability at 24 h using medium light intensity.^45^ Other studies found comparable results, with high viability between 75% and 90%.^33,46^ In this context, for 3D bioprinting applications, GelMA concentrations of <5% (w/v) are suitable for maintaining cell viability with limitations in the geometry fidelity.^46,47^ Concentrations of >15% can increase the viscosity and geometric fidelity that can impact cell viability.^36,48^ Therefore, it is crucial to balance the mechanical properties and biological functionality to achieve superior results using GelMA.

Controlled release of FGF-2 for 30 days showed a burst release in the first week and low levels of release over 30 days. These results were comparable to reported neurotrophic factor release values, demonstrating absorption/binding to the biomaterial surface, allowing FGF-2 to remain available at a stable concentration.^49–51^ A concentration of ∼1 ng mL^–1^ was released from days 7 to 30. Similarly, neurotrophic factors such as glial-cell-line-derived neurotrophic factor (GDNF) and NGF were added at equivalent concentrations (∼1 μg mL^–1^) and released within the range of 1–10 ng mL^–1^, suggesting that this as an optimal concentration range to enhance biological activity and nerve regeneration.^52–54^ Possibly, superior release in a low GelMA concentration can be related to better permeability in low concentration.^51^ Considering its own FGF-2 thermostability of 20 days at 37 °C, we assume that the bioactive effect will be enhanced for 50 days. In addition, the high rate of FGF-2 retained in GelMA will prolong the efficacy due to its release, along with degradation. Importantly, in our study, FGF-2 was maintained for several weeks during the therapeutic window when endogenous sources for nerve regeneration began to decline.^9,10^

Conventionally manufactured NGCs are simple cylindrical structures, and when complex designs are intended, fabrication processes become challenging and less efficient.^21,23^ Here, we developed a hybrid approach using microextrusion 3D printing with inherent buildability, without support structures, and consisting of a PCL outer layer and a GelMA inner layer. The NGCs dimensions were adjusted to match the nerve diameter (<2 mm) to avoid compression and maintain regenerative support, the optimal thickness (100–300 μm), and the size of nerve lesion (8 mm).^16,21^ The dimensions were comparable to polyethylene tubes (BD Intramedic), which have an ID of 1.57 mm, an OD of 2.8 mm, and a wall thickness of 510 μm. Our approach proved to be a practical and reproducible method, with the ability to control the desired geometry.

Experimental models using critical lesions avoid bias related to intrinsic regenerative capacity, and only 10% of axons effectively regenerate into the NGC.^55,56^ Motor recovery by catwalk analysis, contact intensity, and based on support of the hindlimbs was increased in the autograft and NGCs+FGF-2 after 10 weeks. In addition, in the same groups, sensory evaluation showed a recovery of the hyperalgesia intensity, indicating a reduction of neuropathic pain 12 weeks after the experimental procedures. This finding indicates a similar performance between standard treatment and rats treated with NGCs releasing FGF-2. In the same context, upon electrophysiological and morphological evaluation, the autograft and NGCs+FGF2 groups showed better results in latency, duration, and NCV at 12 weeks, indicating the presence of myelinated axons. These findings were correlated with morphological and ultrastructural analysis by TEM and by the presence of positive expression of the myelin specific marker (fluoromyelin). The shorter latency observed in the NGCs+FGF2 and autograft groups, similar to the normal group, suggests the presence of myelinated fibers that improved the conduction time in neuromuscular transmission.^56,57^ The amplitude, which reflects the strength of the action potential, remained reduced across all groups. Despite this, we observed improvements in morphological outcomes including increased axonal diameters. This discrepancy may be attributed to factors such as the lack of muscle response due to fibrosis and atrophy, partial reinnervation, collateral reinnervation, or presence of immature or nonfunctional axons.^57,58^ Therefore, electrophysiological studies beyond 12 weeks may better reflect the regeneration. In rats, the CMAP amplitude in gastrocnemius and tibialis cranialis reaches a plateau by 3–4 months in post-injury.^57^

Improvements in the myelin axon unit diameter, myelin sheath thickness, and *g* ratio measurements in the autograft and NGCs+FGF2 groups compared to NGCs alone were observed. However, the correlation (*g* ratio vs axon diameter) was similar between the autograft, NGCs, and NGCs+FGF2 groups, suggesting that our 3D-printed nerve construct could support regeneration enhanced with bioactive molecules, comparable to previous studies.^23,31^ Using conventional techniques combining fibrin hydrogels and PCL scaffolds, improvements in the histological parameters were observed in short-gap defects.^28,31,59,60^ However, using our approach in long-gap defects, a higher presence of myelinated axons was observed, which was close to that of the contralateral group and equivalent to that of the autograft group. Although previous results showed that NGCs improved regeneration in a long-gap defect, electrophysiological parameters were lower compared to our results.^59,60^

Nerve regeneration is strongly influenced by a pro-regenerative microenvironment.^9,11^ Neurotrophins act selectively on P75NTR receptors expressed in Schwann cells during regeneration, inducing antiapoptotic signals.^61^ A high P75NTR expression level is maintained until contact between the (re)­growing axons and Schwann cells has been established and (re)­myelination initiated.^61^ Immunolabeling results showed a positive expression of P75NTR in the NGCs+FGF2 group at 30 days. In addition, the reactivity of S100 and neurofilament cytoskeleton protein was increased in the NGCs+FGF2 groups 30 days after nerve repair. Similarly, high expression levels of S100 and P75NTR were observed following nerve repair with NGCs loaded with GDNF or stem cells during the regeneration process in PNI with long gaps.^16,28,31,54^ These results suggest the activation of nerve regeneration-related pathways, leading to Schwann cell proliferation, Büngner band formation, laminin deposition, and cytoskeletal reorganization, processes strongly associated with the therapeutic effects of FGF-2.^43^ In comparation, using GDNF, increasing of Schwann cell migration was observed *in vitro*.^62^ However, this effect was not observed *in vivo*, likely due to the low stability of the neurotrophic factor.^26^ When the release of GNDF was controlled using NGCs composed of inorganic minerals and GelMA, Schwann cell proliferation increased, leading to enhanced nerve regeneration.^54^

Our results align with the three main phases of nerve regeneration: (1) axonal regeneration, (2) target reinnervation, and (3) functional recovery (sensorimotor integration).^56^ Our approach corresponds to phase 1, evidenced by axonal sprouting and elongation across the injury site.^56^ The regenerative environment within NGCs, combined with sustained FGF-2 exposure, may activate pathways such as Ras/mitogen-activated protein kinase/extracellular signal-regulated kinase (Ras/MAPK/ERK) and phosphoinositide 3-kinase/protein kinase B (PI3K/Akt), promoting axonal growth, Schwann cell proliferation, reinnervation, synaptic reconnection, and remyelination (2–6 weeks post-injury).^43,56^ This was supported by an increased expression of proteins linked to Schwann cell activity and cytoskeletal organization (S100 and neurofilament), elevated neurotrophin receptor (P75NTR) levels, and positive myelin sheath staining. Notably, P75NTR is upregulated during axon elongation and functional recovery.^43,61^ The increased number of axons and shift toward myelinated fibers observed in morphological analysis correspond to phase 2 (2–6 weeks postinjury), aligning with the sustained, lower-concentration release of FGF-2 during the 30 days after the initial burst. Subsequently, gradual improvements in nerve conduction, reduced latency, and motor and nociceptive functions were observed, consistent with phase 3 (2–16 weeks postinjury), indicating reinnervation and maturation of axons and myelin. Together, these findings suggest that the NGCs created a proregenerative microenvironment that could be optimized by the long-term release of hyperstable FGF-2 beyond 16 weeks.

NGCs must provide sustained structural support during both early and chronic phases of regeneration and subsequently degrade to avoid long-term foreign body responses.^5^ Although 10% GelMA degraded within 6 days *in vitro*, residual material was observed via TEM *in vivo*, indicating partial but prolonged persistence without eliciting any substantial foreign body response. Similar studies reported rapid *in vitro* degradation with collagenase (1–2 days), while *in vivo* degradation after PNI occurred more slowly over 1–3 months without hindering axonal regeneration.^17,32,63^ Additionally, GelMA and PCL scaffolds remained detectable 12 weeks postimplantation without impairing brain or nerve tissue regeneration.^23,64^ PCL degrades slowly via hydrolysis of ester bonds, often taking 2–3 years for high-molecular-weight forms, which limits the accurate assessment of its degradation rate in rat implantation studies.^23,65,66^ However, this slow degradation aligns with human nerve regeneration timelines because proximal nerve injuries in the upper limbs may take 2–3 years for full reinnervation at ∼1 mm day^–1^.^5^ Our implanted NGCs showed no macroscopic evidence of rejection with adequate integration into the surrounding tissues.

Microscopic evaluation herein suggests enhanced macrophage activation in the NGC groups. In this regard, one limitation of this study is the lack of a direct assessment of macrophage polarization. However, to assess such macrophage response, different approaches, such as flow cytometry, would be necessary, which was out of the scope of the present work.^67,68^ Future studies exploring the dynamic modulation of macrophage phenotypes could help to elucidate the underlying mechanisms. Polarized macrophages—M1 (proinflammatory), M2 (antiinflammatory), and intermediate subtypes (M2a–d)—act in a coordinated manner to clear myelin, axonal debris, and inhibitory signals, promote vascularization, and remodel the extracellular matrix.^28,69,70^ Another point of concern is that the *in vivo* model does not fully recapitulate the complexity of human nerve regeneration. Long-term follow-up in long-gap injury models, including larger mammals and even primates, may better reflect axon maturation, remyelination, and functional recovery. Different *in vivo* degradation of the biomaterials also warrants studies aimed at fine-tuning the degradation profiles of GelMA and PCL in relation to nerve repair. Last, while hyperstable FGF-2 showed promising effects, further optimization of its dosing, release kinetics, and pharmacodynamic profile is needed.

## Conclusion

In our study, a bilayer hybrid NGCs composed of PCL/GelMA integrated with thermostable FGF-2 was developed for the regeneration of severe PNIs. The biofabrication strategy employed a system in which PCL supported the channel structure, and GelMA exhibited bioactivity with controlled release of FGF-2 with features such as customization, rapid fabrication, biocompatibility, and biomimetic properties. These features optimized a regenerative microenvironment through the guidance of regenerating axons, myelination, Schwann cell proliferation, and an increased expression of neurotrophic factors receptor (P75NTR). The results demonstrate that NGCs fabricated using a combination of photopolymerizable hydrogels and biocompatible thermoplastic synthetic polymers with controlled drug release have significantly improved functional, motor, electrophysiological, and histological regeneration after experimental sciatic nerve injury in rats. This NGCs technology shows potential for translation into clinical trials for severe PNIs.

## Supplementary Material



## Data Availability

Data will be made available upon request.

## Notes

Determination of the DoF, photocross-linking efficiency, and pH characterization, porosity characterization, indirect cytotoxicity analysis of GelMA hydrogels, direct cytotoxicity analysis of GelMA hydrogels, printing capacity and resolution of PCL polymer, swelling and enzymatic degradation of GelMA hydrogels, and PCL/GelMA 3D-printed NGCs for sciatic nerve regeneration (PDF)

## Abbreviations

3D: tridimensional
BDNF: brain-derived neurotrophic factor
CAD: computer aided design
CMAPs: compound muscle action potentials
CNS: central nervous system
CNTF: ciliary neurotrophic factor
DMEM: Dulbecco’s modified eagle medium
DoF: degree of functionalization
ED: external diameter
EMG: electromyography
FBS: fetal bovine serum
FGF-2: fibroblast growth factor 2
FGF: fibroblast growth factor
*G*″: loss modulus
*G*′: storage modulus
GAP-43: growth-associated protein 43
GAPDH: glyceraldehyde 3-phosphate dehydrogenase
GDNF: glial-cell-line-derived neurotrophic factor
GelMA: gelatin methacryloyl
HBSS: Hank’s balanced salt solution
H&E: hematoxylin and eosin
IBA-1: ionized calcium-binding adaptor molecule 1
ID: internal diameter
MSCs: mesenchymal stem cells
NCV: nerve conduction velocity
NGCs: nerve guidance conduits
NGF: nerve growth factor
NT-3: neurotrophin 3
NT-4: neurotrophin 4
NT-5: neurotrophin 5
P75NTR: p75 neurotrophin receptor
PI3K/Akt: phosphoinositide 3-kinase/protein kinase B
PCL: polycaprolactone
PNI: peripheral nerve injuries
PNS: peripheral nervous system
RAGs: regeneration-associated genes
Ras/MAPK/ERK: Ras/mitogen-activated protein kinase/extracellular signal-regulated kinase
S100: S100 calcium binding protein
SEM: scanning electron microscopy
SoC: standard of care
tan δ: damping factor
TEM: transmission electron microscopy
VEGF: vascular endothelial growth factor
ω: angular frequency
