# Supplementary material for: Long-Gap Sciatic Nerve Regeneration Using 3D-Printed Nerve Conduits with Controlled FGF‑2 Release
Source: ACS Appl Mater Interfaces. 2025 Jul 7;17(28):40237–57. doi: 10.1021/acsami.5c08237 (PMC12278209; doi:10.1021/acsami.5c08237)
Supplement: Supplementary file 1 [file am5c08237_si_001.pdf]

# SUPPORTING INFORMATION

# Long-gap sciatic nerve regeneration using 3D-printed nerve conduits with controlled FGF-2 release

*Diego N. Rodriguez-Sanchez*[ab], *Leticia A.M. de Carvalho*[a], *Ingri Mancilla-Corzo*[c], *Luciana P. Cartarozzi*[a], *Saeed Safari*[b], *Menekse Ermis*[b], *Marcos A. d'Ávila*[c], *Alexandre L.R. Oliveira*[a*]

[a]Department of Structural and Functional Biology, Institute of Biology, University of Campinas (UNICAMP), Laboratory of Nerve Regeneration, Campinas, Sao Paulo, 13083-970, Brazil.

[b]Terasaki Institute for Biomedical Innovation (TIBI), Woodland Hills, California, 91367, USA.

[c]Department of Manufacturing and Materials Engineering, School of Mechanical Engineering, University of Campinas (UNICAMP), Campinas, Sao Paulo, 13083-860, Brazil.

[*]Corresponding author: Alexandre Leite Rodrigues de Oliveira, Ph.D. E-mail: alroliv@unicamp.br

***Determination of the degree of functionalization (DoF).*** The ninhydrin assay was used to detect free primary amines following the gelatin functionalization process. A standard curve was prepared by serially diluting unmodified gelatin in PBS to concentrations ranging from 0 to 10 mg $mL^{-1}$. Functionalized GelMA samples were plated in triplicate without dilution. Ninhydrin solution in ethanol (2.2 mg $mL^{-1}$) was added to each sample at a 1:8 (v/v) ratio relative to the gelatin solution (total volume of 100 $\mu$L per well), and the plates were incubated at 70 ˚C for 30 minutes. Absorbance was measured at 570 nm, and the fraction of available amine groups in each functionalized sample was calculated using the following equation:

$$Avaliable\ amine\ fraction: \frac{Apparent\ concentration}{nominal\ concentration} \quad \text{Equation S1}$$

Where the apparent concentration was obtained by comparison with the standard curve. The percentage (%) of degree of functionalization (DoF) was determined using the following equation:

$$DoF\ (\%): 100\ x \left(1 - \frac{apparent\ concentration\ (sample)}{nominal\ concentration\ (sample)}\right) \quad \text{Equation S2}$$

***Photocrosslinking efficiency.*** Samples of GelMA at concentrations of 2.5% 5% and 10% (wt/vol) were used, maintaining a constant concentration of 0.5% (wt/vol) of the photoinitiator IC2959. After preparing the samples these were deposited into transparent silicone molds in rectangular shape (7mm x 14mm x 4mm) (Sigma-Aldrich, Burlington, MA, USA) and subsequently exposed to UV light using a 365nm lamp (E19UV, Wuben, Guangdong, China) at a height of 45 mm to form GelMA constructs (18 $mW/cm^2$). Five UV exposure times were defined: 40, 100, 200, 240, and 300 seconds, respectively. Rectangular GelMA hydrogels constructs were obtained, and longitudinal stability was observed at rest and after ~45° inclination.

***pH characterization:*** The *pH* evaluation of 2.5%, 5% and 10% GelMA samples photopolymerized for 200 seconds was conducted. GelMA constructs were incubated in triplicate in sterile 24-well polystyrene plates (Nest Biotech, Wuxi, China) and supplemented with low glucose DMEM without pyruvate, glutamine, and phenol red (Gibco, Sao Paulo, SP, Brazil) for 24, 48, 72, and 120 hours. The control consisted of DMEM alone. After the defined periods, *pH* was semi-quantitatively assessed using colorimetric strips (*pH* 0 -14, Kasvi, Guangzhou, China).

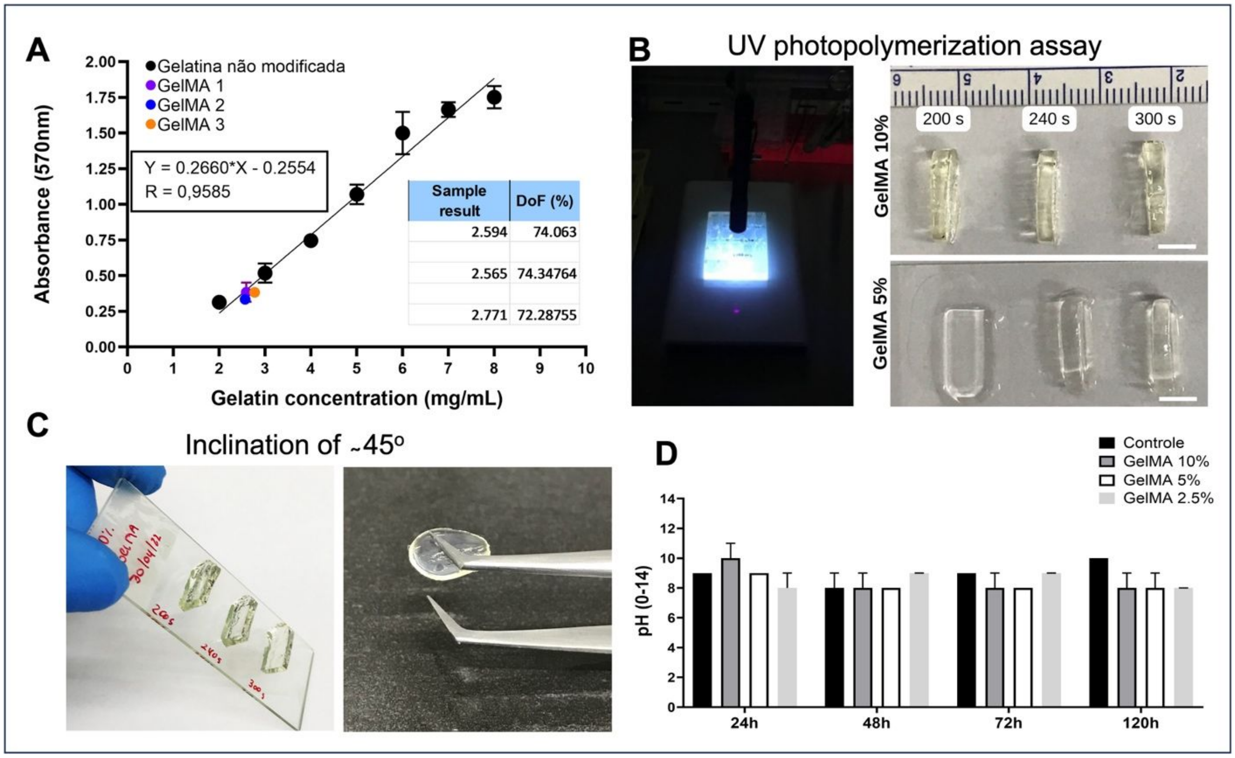


**Figure S1.** Determination of the degree of functionalization (DoF), photocrosslinking efficiency, and *pH* characterization of GelMA samples. (A) The standard curve demonstrated linear absorbance of gelatin between 2-8 mg $mL^{-1}$ (R = 0.9585; $p < 0.0001$). GelMA samples batches exhibited a DoF (%) between 72-74 (%). (B) UV photopolymerization test were performed using 5% and 10% (wt/v) GelMA. (B) Setup of irradiation with a UV 365 nm light source. Fine edges and structural fidelity are observed from 200 seconds with GelMA 10%. Scale bar: 5mm. (C) Stability of GelMA 10% after inclination and disc formation of GelMA 10%. (D) Graph showing

*pH* values measured at 24, 48, 72, and 120 hours after incubation ($p > 0.05$). All analyses: $n = 3$. Values are represented as mean ± SEM. $p < 0.0332$*, $p < 0.0021$**, $p < 0.0002$***, $p < 0.0001$****.

***Porosity Characterization.*** GelMA hydrogels (2.5 %, 5 % and 10%) (wt/v) were UV-crosslinked (12 mW/cm$^2$, 240 seconds), freeze-dried for 12 hours, and placed on scanning electronic microscopy (SEM) stubs using double-sided carbon tape. Then, the samples were coated with gold for 200 seconds using a sputter coater (Balzers, SCD-050, Liechtenstein) at 20W, during 200s. The microstructure of dried composites hydrogels was evaluated by a SEM (TESCAN MIRA 4, Brno, Czech Republic) at 15 kV, and obtained percentage of microporosity ($n = 5$) and analyzed using a particles analysis tool from Image J (NIH, Bethesda, USA).

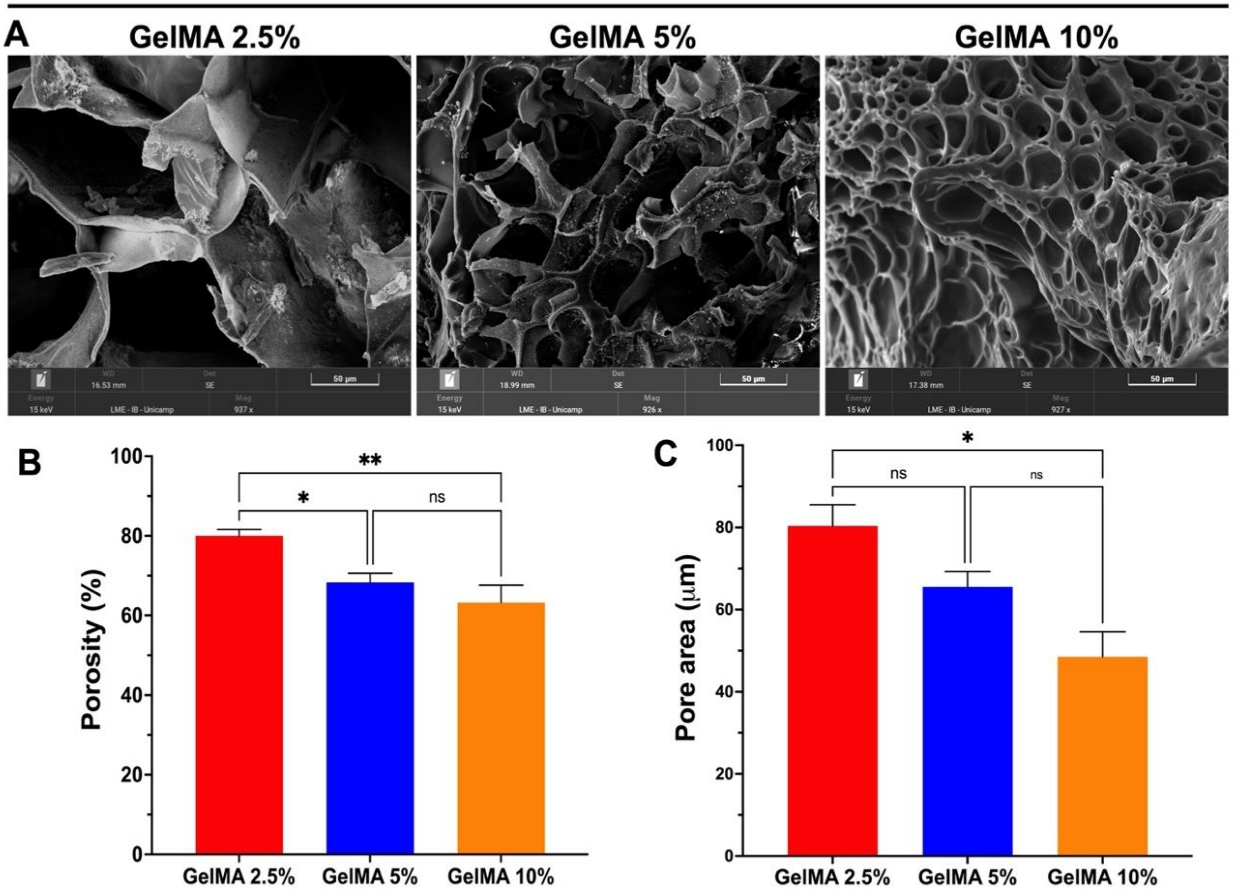


**Figure S2.** Characterization of Porosity in GelMA Hydrogels and NGCs. (A) High-resolution SEM images show a decrease in internal porosity with increasing GelMA concentrations from 2.5% to 10% (wt/v) after photocrosslinking. Quantitative analysis of porosity percentage per area (B) and pore area (C) confirms this trend to porosity reduction across 2.5%, 5%, and 10% (wt/v) GelMA. Data are presented as mean ± SEM. $p < 0.0332$*, $p < 0.0021$**, $p < 0.0002$***, $p < 0.0001$****. All analyses: n = 5. Scale bars: 50 $\mu$m and 100 $\mu$m.

***Indirect Cytotoxicity analysis of GelMA hydrogels.*** Indirect cytotoxicity of GelMA hydrogels (2.5%, 5%, and 10%) containing FGF-2 (2 $\mu$g $mL^{-1}$) was evaluated in S16 rat Schwann cells (ATCC CRL-2941). Cells were exposed to UV-crosslinked GelMA (18 $mW/cm^2$ for 240 seconds), and viability was assessed at days 1, 3, and 5 using a Live/Dead assay (Thermo Fisher L3224).

After each time point, cells were stained with 1 μg $mL^{-1}$ Calcein-AM and 2 μg $mL^{-1}$ ethidium homodimer for 30 minutes. Fluorescence images (n = 3 /time point) were captured using an Echo Revolve microscope and analyzed with ImageJ (NIH) particle analysis. Viability (%) was calculated as follows:

$$Viability\ (\%): \frac{Number\ of\ cells\ live}{Total\ number\ of\ cells}\ x\ 100 \qquad \text{Equation S3}$$

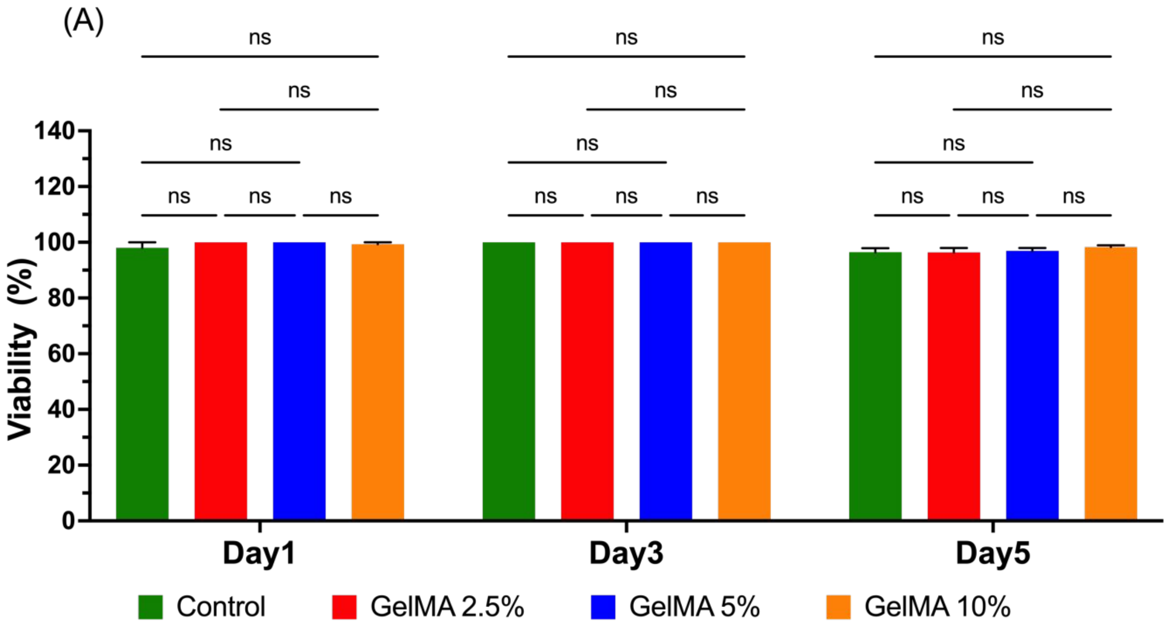


**Figure S3.** Viability analysis of S16 cells exposed to photocrosslinked GelMA hydrogels. (A) Percentage of viable cells at days 1, 3, and 5 following exposures to 2.5%, 5%, and 10% (w/v) GelMA hydrogels. All analyses: n = 3. Data are presented as mean ± SEM. $p < 0.0332$*, $p < 0.0021$**, $p < 0.0002$***, $p < 0.0001$****.

***Direct Cytotoxicity analysis of GelMA hydrogels.*** Human dental pulp mesenchymal stem cells (MSCs) (Poietics™, Lonza, Walkersville, MD, USA) at a concentration of $1 \times 10^6$ cells $mL^{-1}$ were encapsulated in 5% and 10% GelMA, which demonstrated better mechanical properties. The

mixture was then transferred into transparent silicone molds (7 mm x 14 mm x 4 mm) (Sigma-Aldrich, Burlington, MA, USA) and UV crosslinked (18 mW/cm$^2$, 240 seconds). The hydrogels were maintained under standard culture conditions with DMEM low-glucose (90%), 10% fetal bovine serum (FBS), and 1% penicillin (10,000 IU mL$^{-1}$) and streptomycin (10 mg mL$^{-1}$) (all from Gibco). Viability/cytotoxicity was assessed using a Live/Dead stain at 2, 7, and 14 days, with triplicate samples.

Morphology and metabolic activity of DPMSCs were verified through using a mitochondrial cell marker. GelMA 5% and 10% constructs containing DPMSCs were fixed in 4% paraformaldehyde (PFA) and immersed in sucrose and included in Tissue-Tek OCT. Cryosections 16 $\mu$m sections were prepared. During the immunofluorescence reaction, slides were washed with 0.01 M PB 3 times and blocked (3% BSA in 0.1 M PB) for 45 minutes. They were incubated with primary antibody anti-human mitochondria (ab92824, Abcam, Cambridge, UK) for 2 hours. Sections were washed with 0.01 M PB and mounted in glycerol/PB (3:1). Samples were observed under a fluorescence microscope (Leica DMB5500, Wetzlar, Germany) and documented with a digital camera (Leica DFC 345 FX) using specific filters.

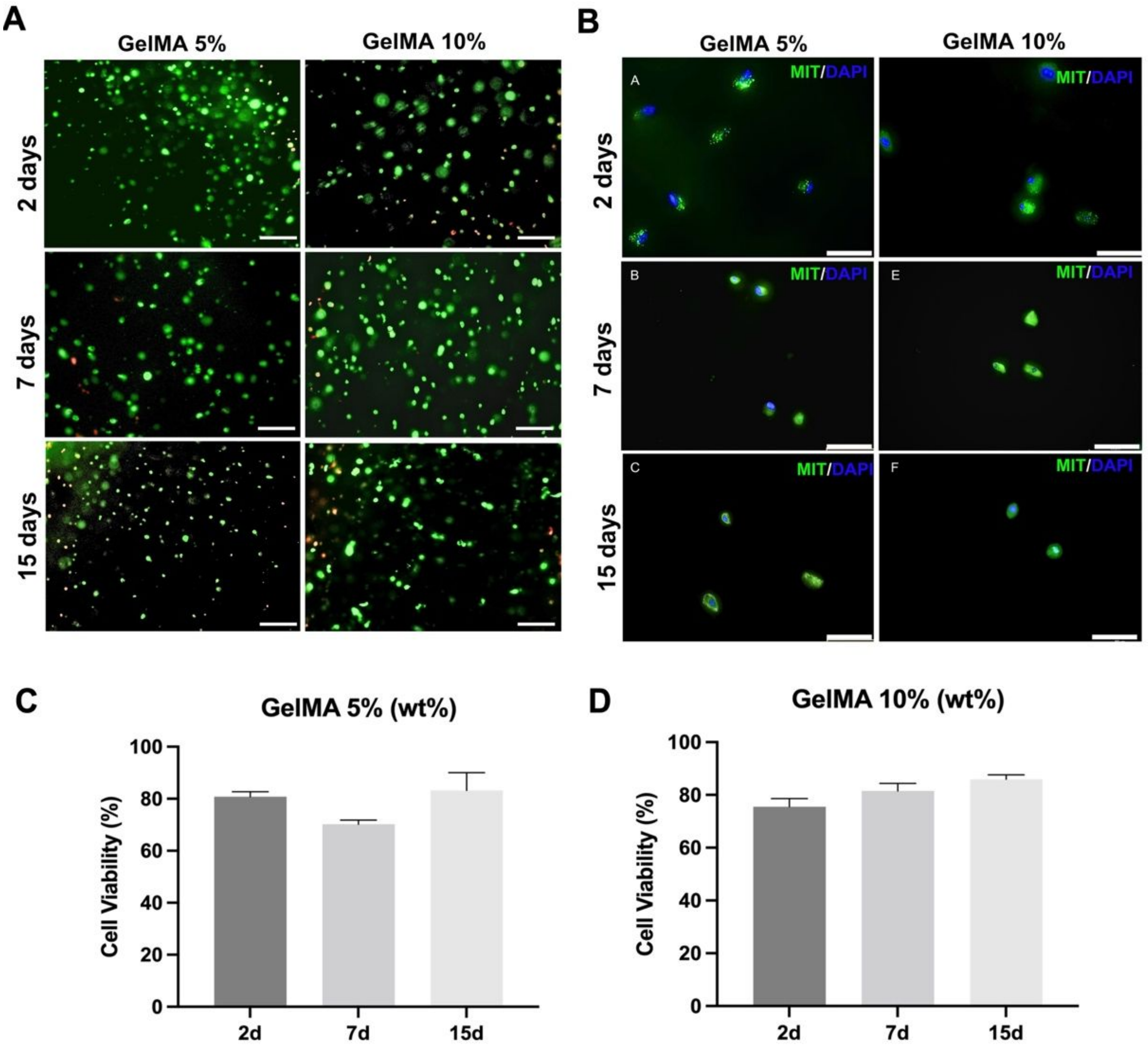


**Figure S4**. Evaluation of direct cytotoxicity and mitochondria expression after human MSCs encapsulation in GelMA hydrogels. (A) Representative image of GelMA 5% encapsulation with live cells stained in green (calcein AM) and dead cells stained in red (ethidium homodimer) at 1, 7 and 15 days. (B) Representative images of cells immunoassayed for human mitochondria in GelMA 5% and 10% (wt/v) after photo crosslinking, during 1, 7 and 15 days. Percentage of cell viability at 2, 7, and 15 days in 5% (C) and 10% (D) GelMA. All analyses: n = 3. Scale bars: 20 $\mu$m and 50 $\mu$m. Values are represented as mean ± SEM. $p < 0.0332$*, $p < 0.0021$**, $p < 0.0002$***, $p < 0.0001$****.

**Printing capacity and resolution of polycaprolactone (PCL) polymer**. Flat scaffolds with dimensions of 10 mm × 10 mm × 0.5 mm were designed using computer-aided design (CAD) software for 3D printing. The scaffolds were fabricated by microextrusion-based 3D printing of continuously melted PCL micro pellets (Mn 50,000) using a thermoplastic printhead on a Bio X (3rd Generation) bioprinter (Cellink-BICO, Gothenburg, Sweden). The printing parameters were as follows: a stainless-steel nozzle with a diameter of 400 µm, extrusion pressure of 180-200 kPa, printhead temperature of 180-200 °C, print bed temperature of 10 °C, print velocity of 7-10 mm $s^{-1}$, layer height of 0.39 mm (100%), and 55% infill with a rectilinear pattern. Printability was assessed through SEM micrographs (n = 8/sample) obtained from PCL 2D scaffolds (n = 3) to optimize printing fidelity. Additionally, pore height, pore width (pore length), and filament diameter of the PCL constructs (n=3) were analyzed using ImageJ software (NIH, Bethesda, USA). The printability (Pr) was determined following the equation:

$$Pr = \frac{\pi}{4C} = \frac{L2}{16A} \qquad \text{Equation S4}$$

Where $A$ represents the pore area and $L$ the pore perimeter. For a perfect square, the Pr equals 1. A value of Pr > 1 indicates over-gelation (or excessive material spreading), while Pr < 1 suggests under-gelation (or insufficient material deposition). Values in the range of 0.9 to 1.1 are generally considered acceptable for 3D printing in tissue engineering applications.

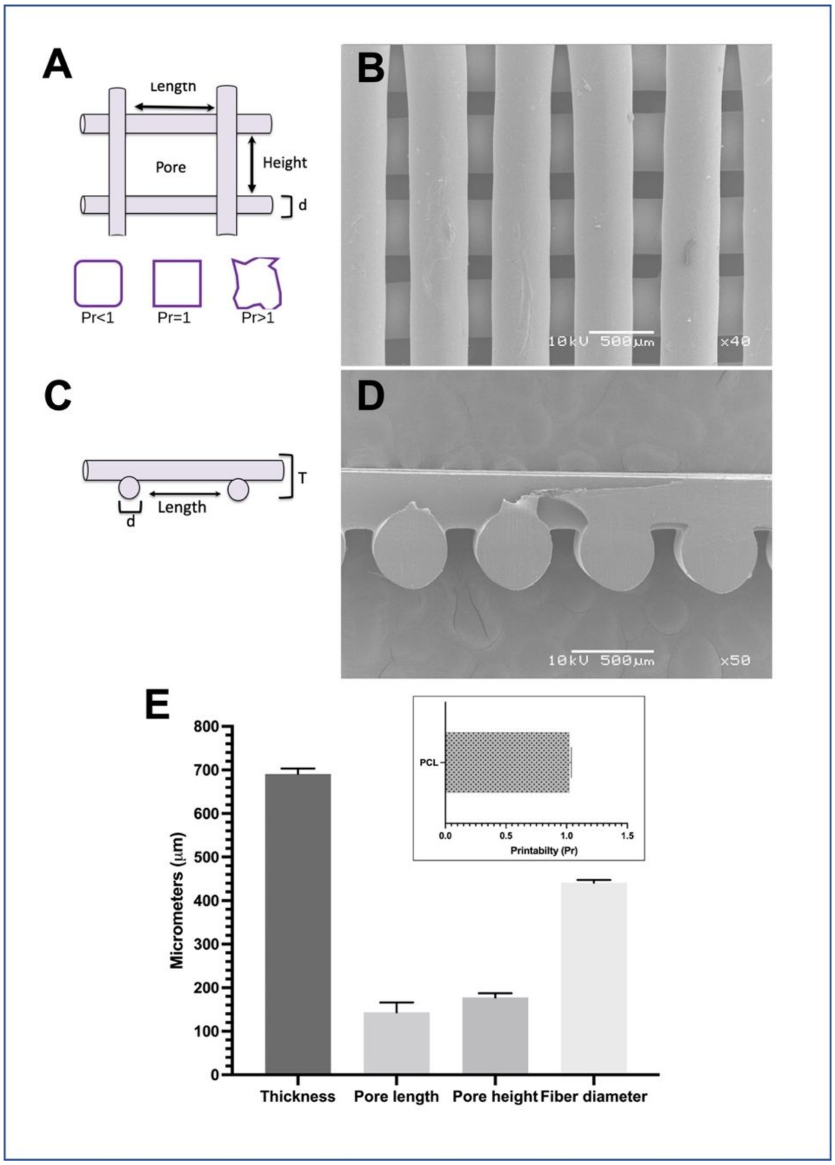


**Figure S5. Analysis of the resolution and printability of the PCL polymer.** (A) Schematic representation of the dorsal view of the printing path and evaluated parameters. (B) Corresponding SEM images of the scaffolds after 3D printing. (C) Schematic representation of the lateral view of the printing path and analyzed parameters. (D) Corresponding SEM images showing scaffold thickness (690.9 ± 4.71 $\mu$m). (E) Analysis of measurements obtained from SEM images showed that the filaments, with a diameter of 441.2 ± 11.01 $\mu$m, were continuously deposited in a square pattern, resulting in the formation of a bilayer membrane. The spaces between the filaments formed

pores with a height of 143.3 ± 39.10 $\mu$m and a width of 177.5 ± 16.40 $\mu$m (n = 3). Data are presented as mean ± SEM. Scale bars: 500 $\mu$m.

**Swelling and Enzymatic degradation of GelMA hydrogels.** The degradation of GelMA hydrogels (2.5%, 5%, and 10% wt/v) was assessed using collagenase type I (1 U $mL^{-1}$; C2674, Sigma-Aldrich, São Paulo, Brazil) at 37 ˚C. UV-crosslinked hydrogels (100 µL; 12 mW/cm², 240 seconds) were placed in pre-weighed containers and incubated in DPBS at 37 ˚C until equilibrium was reached (n = 4). After 24 h, DPBS was replaced with collagenase solution, and samples were incubated further. At predetermined time points, hydrogels were removed, weighed, and fresh collagenase was added to monitor degradation. Degradation was calculates using the following equation:

$$Mass\ loss\ (\%): \frac{Wpre{-}incubation - Wpost{-}incubation}{Wpre{-}incubation}\, x\, 100 \qquad \text{Equation S5}$$

Swelling was determined by measuring the hydrated weight (Ws) after incubating the samples in DPBS at 37 ˚C for 24 hours, followed by gentle blotting and weighing. Dry weight ($W_0$) was obtained after air-drying. The swelling ratio was calculated according to the following equation:

$$Swelling\ ratio\ (\%): \frac{Ws - Wo}{Wo} \qquad \text{Equation S6}$$

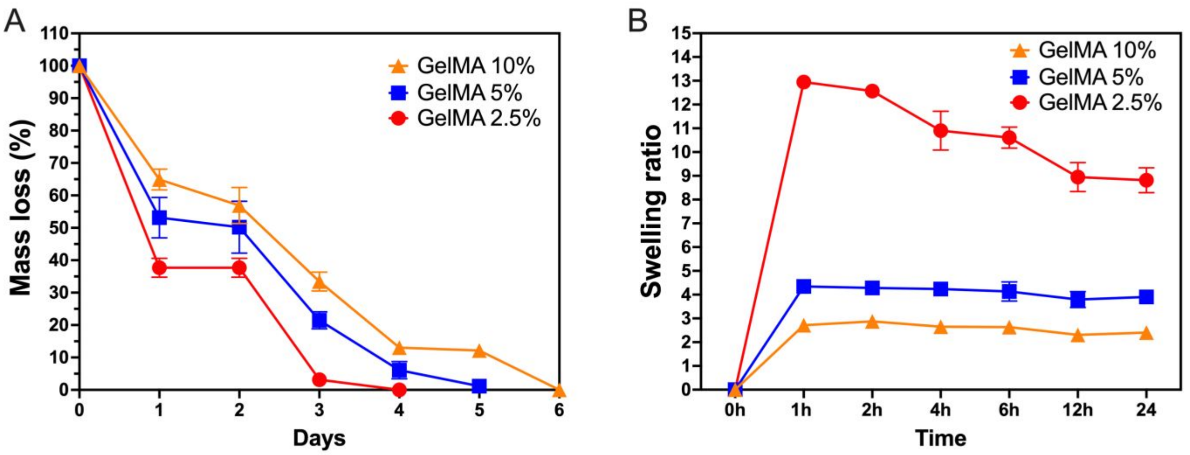

**Figure S6. In vitro degradation and swelling behavior of GelMA hydrogels.** (A) Enzymatic degradation profile of UV cross-linked GelMA hydrogels under in vitro conditions. (B) Swelling ratio of UV cross-linked GelMA hydrogels over time. Data are presented as mean ± SEM.

**Morphological evaluation.** Hematoxylin and eosin (H&E) staining was performed on longitudinal cryosections (12 $\mu$m) from the Autograft, NGC, and NGC+FGF2 groups after nerve repair. Sections were acclimated to room temperature for 30 minutes, followed by hydration in water. They were then immersed in hematoxylin for 5 seconds, washed in water for 2 minutes, and counterstained with eosin for 55 seconds. The staining procedure was finalized with successive immersions in graded alcohol baths (95%, 100% I, 100% II) and xylene. Slides (n = 3 per group) were analyzed using a Leica DM5500B microscope (Leica Microsystems, Wetzlar, Germany).

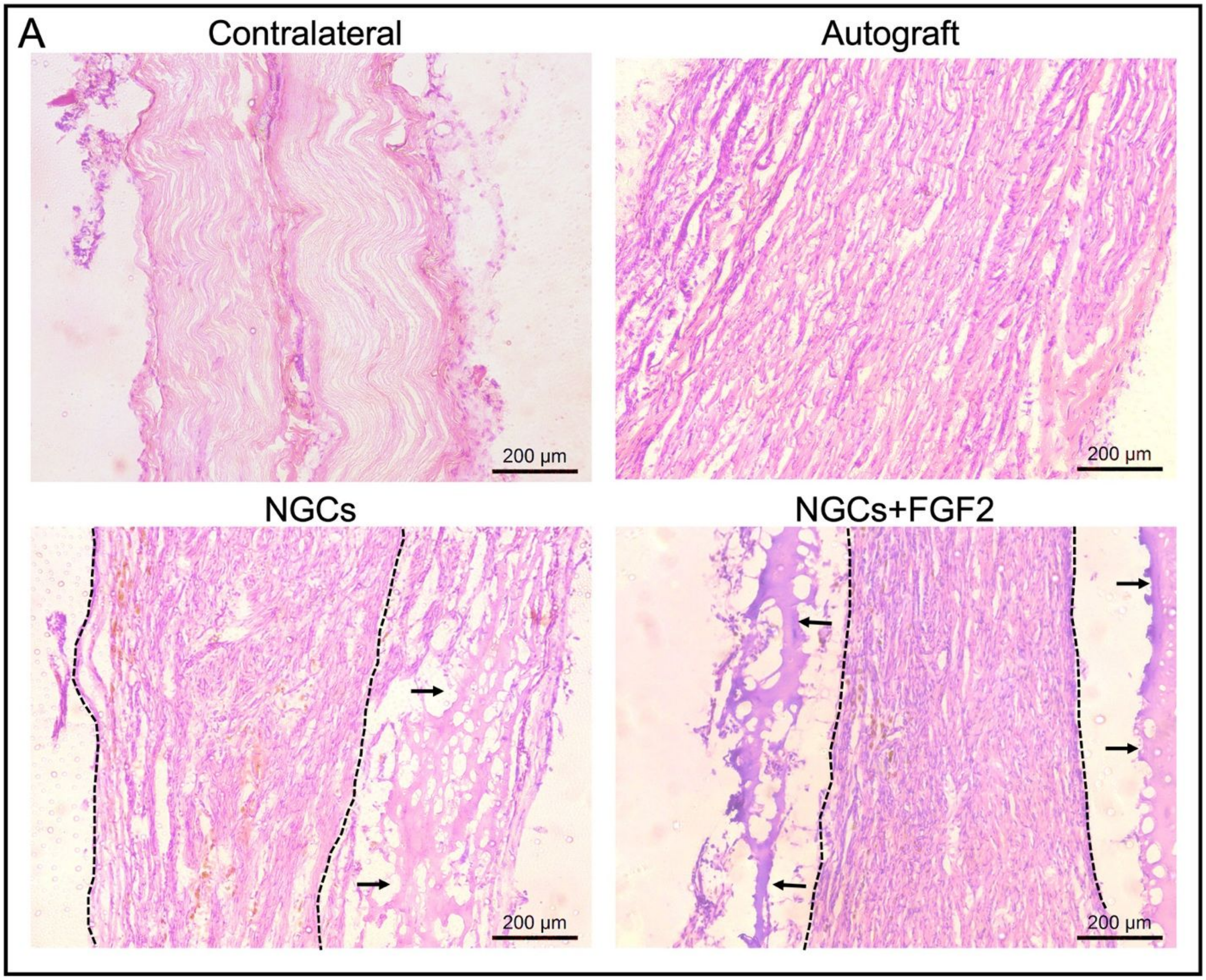


**Figure S7**. PCL/GelMA 3D-printed nerve guidance conduits for sciatic nerve regeneration. (A) Representative images with hematoxylin and eosin (H&E) staining of the mid-section of regenerated sciatic nerves 12 weeks post-NGC implantation. The PCL external wall was previously removed. The remaining GelMA internal wall is marked by black arrows. Scale bars: 200 $\mu$m.
